# Transcription termination and readthrough in African swine fever virus

**DOI:** 10.3389/fimmu.2024.1350267

**Published:** 2024-03-13

**Authors:** Gwenny Cackett, Michal Sýkora, Raquel Portugal, Christopher Dulson, Linda Dixon, Finn Werner

**Affiliations:** ^1^ Institute for Structural and Molecular Biology, University College London, London, United Kingdom; ^2^ Pirbright Institute, Pirbright, Surrey, United Kingdom

**Keywords:** African swine fever virus (ASFV), transcription termination, transcriptomics, RNA polymerase, transcription readthrough, long-read sequencing, Oxford Nanopore

## Abstract

**Introduction:**

African swine fever virus (ASFV) is a nucleocytoplasmic large DNA virus (NCLDV) that encodes its own host-like RNA polymerase (RNAP) and factors required to produce mature mRNA. The formation of accurate mRNA 3′ ends by ASFV RNAP depends on transcription termination, likely enabled by a combination of sequence motifs and transcription factors, although these are poorly understood. The termination of any RNAP is rarely 100% efficient, and the transcriptional “readthrough” at terminators can generate long mRNAs which may interfere with the expression of downstream genes. ASFV transcriptome analyses reveal a landscape of heterogeneous mRNA 3′ termini, likely a combination of *bona fide* termination sites and the result of mRNA degradation and processing. While short-read sequencing (SRS) like 3′ RNA-seq indicates an accumulation of mRNA 3′ ends at specific sites, it cannot inform about which promoters and transcription start sites (TSSs) directed their synthesis, i.e., information about the complete and unprocessed mRNAs at nucleotide resolution.

**Methods:**

Here, we report a rigorous analysis of full-length ASFV transcripts using long-read sequencing (LRS). We systematically compared transcription termination sites predicted from SRS 3′ RNA-seq with 3′ ends mapped by LRS during early and late infection.

**Results:**

Using *in-vitro* transcription assays, we show that recombinant ASFV RNAP terminates transcription at polyT stretches in the non-template strand, similar to the archaeal RNAP or eukaryotic RNAPIII, unaided by secondary RNA structures or predicted viral termination factors. Our results cement this T-rich motif (U-rich in the RNA) as a universal transcription termination signal in ASFV. Many genes share the usage of the same terminators, while genes can also use a range of terminators to generate transcript isoforms varying enormously in length. A key factor in the latter phenomenon is the highly abundant terminator readthrough we observed, which is more prevalent during late compared with early infection.

**Discussion:**

This indicates that ASFV mRNAs under the control of late gene promoters utilize different termination mechanisms and factors to early promoters and/or that cellular factors influence the viral transcriptome landscape differently during the late stages of infection.

## Introduction

Evolutionary conserved double-psi beta-barrel (DPBB) RNA polymerases (RNAPs) transcribe the genomes of bacteria, archaea, and eukaryotes (1). Eukaryotic double-stranded DNA viruses including nucleocytoplasmic large DNA viruses (NCLDVs) like variola virus (smallpox), vaccinia virus (VACV), and African swine fever virus (ASFV) also employ a DPBB RNAP to transcribe the viral genome in the cytoplasm of the infected cell. In comparison to their cellular counterparts, the viral transcription systems are understudied and their RNAPs and associated factors are poorly understood, despite their importance as therapeutic targets in the treatment of viral disease. ASFV causes hemorrhagic fever in domestic and wild pigs with almost 100% fatality, and as there are no available antiviral drugs or vaccines, it presents a severe threat to global food security. As seen in other NCLDVs, ASFV particles include all components required for early virus transcription including RNAP, regulatory factors, capping enzyme, and polyadenylate polymerase (2, 3). The accurate formation of the mRNA 5′ end relies on events during initiation, i.e., transcription start site (TSS) selection, which is dependent on the RNAP, initiation factors, and the ASFV promoter sequences we previously identified (4). In comparison to initiation, transcription termination is poorly understood, despite being an essential aspect of gene expression control. However, most terminators across all RNAP transcription systems are “leaky” to some extent and will allow the “readthrough” of some RNAPs into regions downstream of a terminator. In eukaryotes, this phenomenon is often associated with cellular stress and viral infection (5–7). Importantly, premature termination in the upstream region of genes provides a potent means for regulating the transcription output in eukaryotes, archaea, and bacteria. Prokaryotic genomes can be organized into multicistronic operons, where several ORFs are under the control of the same promoter and transcribed as one mRNA, and premature termination is an effective means of modulating the stoichiometry of gene products. Though in the dense prokaryotic genomes of bacteria and archaea, there are both factor-dependent and intrinsic termination mechanisms employed to prevent disruptive readthrough into closely neighboring genes (8, 9). In summary, transcription termination is not only facilitating precise mRNA 3′ end formation and polyadenylation but also a means for gene regulation.

Most cellular RNAPs from bacteria, archaea, and eukaryotes utilize “intrinsic” or factor-independent means of termination that involves a polyT stretch in the coding strand, equivalent to a polyU tract in the mRNA (10–12). Bacterial intrinsic terminators furthermore include an RNA hairpin secondary structure element upstream of the ~8-nt polyU tract. Termination in *Escherichia coli* can be enhanced or suppressed by accessory factors including NusA, though it is not essential for termination (13). PolyU tracts also facilitate termination by RNAPIII and the archaeal RNAP, but the latter is not dependent on any secondary structures. Phylogenetic analysis of the large RNAP subunits across NCLDVs, vRPB1 and vRPB2, revealed that *Asfarviridae* including ASFV RNAP are most closely related to RNAPI (14). This raises the possibility that ASFV termination also resembles that of RNAPI, which utilizes the Reb1p factor in conjunction with the recognition of a T-rich sequence motif required for transcript cleavage, release, and processing of the nascent transcript (15–18). Termination depends on the RNAPI subunit Rpa12p which is conserved in some NCLDVs like ASFV (vRPB9) but not VACV (15, 16, 19, 20). In contrast to prokaryotes, archaea, and bacteria, termination by eukaryotic RNAPII is coupled to polyadenylation, and cellular stress impairs termination, particularly on genes with a weaker polyadenylation signal (PAS) sequence (21, 22). Interestingly, both RNAPII and VACV utilize a PAS, AAUAAA/AUUAAA and UUUUUN, respectively, which are located upstream of the site at which the RNA terminates (10–30 nt and ~40 nt, respectively) (22–24). For RNAPII, this involves processing by endonucleolytic cleavage and polyadenylation. The process in poxviruses is far less understood than its eukaryotic hosts but is thought to involve a range of factors that change as the stage of infection progresses. Despite the different predicted termination signals in *Poxviridae* and *Asfarviridae*, several transcription termination factors are conserved between them (**Table 1**).

**Table 1:** Summary of predicted transcription VACV termination factor homologs conserved in ASFV, their function, and their presence or absence in virus particles.

ASFV gene	In ASFV particles (3)	VACV factor name	VACV gene	In VACV particles (25)	Role in VACV transcription
*NP868R* (26)	Yes	VTF/CE	*D1R* and *D12L*	Yes	All—transcript capping and termination (27) cVACV–RNAP complex (20)
*Q706L* (28)	Yes	NPH-I	*D11L*	Yes	All—termination (29) cVACV–RNAP complex (20)
–	–	VLTF-4	*H5R*	Yes	Intermediate/late transcription elongation/termination (30)
–	–	G2	*G2R*	No	Intermediate/late transcription elongation/termination (31)
*QP509L* | *A859L* (32–35)	No | No	A18	*A18R*	Yes	DNA helicase, intermediate and late transcription termination factor (36, 37)
*B962L* (32)	Yes	NPH-II	*I8*	Yes	RNA-dependent NTPase, DE-H family (38)

Improving the characterization of the mRNA 3′ landscape is a vital step toward understanding transcription termination and mRNA processing in ASFV. We previously explored the ASFV transcriptome including short-read sequencing (SRS) Illumina-based 3′ RNA-seq in the first genome-wide analysis of transcription termination in ASFV (4). This method, however, had limitations in detecting 3′ end signals from the RNAs of late genes and crucially no way to distinguish signals arising from transcription readthrough. Considering the high variability of ASFV transcript lengths from previous individual gene studies (39–59), the best method for investigating ASFV RNA 3′ end formation is long-read sequencing (LRS), such as sequencing using Oxford Nanopore Technologies (60) as demonstrated in the seminal work by Olasz et al. (61) and Torma et al. (62). We, therefore, followed up the previous SRS analysis of transcription termination in ASFV-BA71V by applying an LRS strategy, to specifically investigate the utilization of termination sites during early and late stages of an ASFV infection time course, with a focus on sequence motif utilization and the frequency of terminator readthrough. We found that from early to late infection, there was a marked increase in readthrough at transcription terminators. Besides the stage of viral gene expression, the relative orientation of genes to their neighbors appears to influence termination. Terminator readthrough is common among ASFV genes, but reduced by longer polyT stretches, and for convergent gene pairs (genes oriented head-to-head), we observed disruptive premature 3′ end formation. Using a highly defined *in-vitro* transcription system, we show that a recombinant ASFV core RNAP is able to terminate faithfully at polyT terminators in the absence of termination factors like NPH-I, which plays an important role in termination for vaccinia virus RNAP (29). ASFV RNAP termination is furthermore independent of RNA secondary structures upstream of the polyT, a hallmark of bacterial intrinsic terminators (11). This emphasizes the conservation of termination mechanisms between ASFV RNAP, archaeal RNAP (10), and RNAPIII (12, 63, 64)—between viruses and cellular domains of life.

## Results

### Comparison of short- and long-read sequencing in ASFV

**Figure 1:**
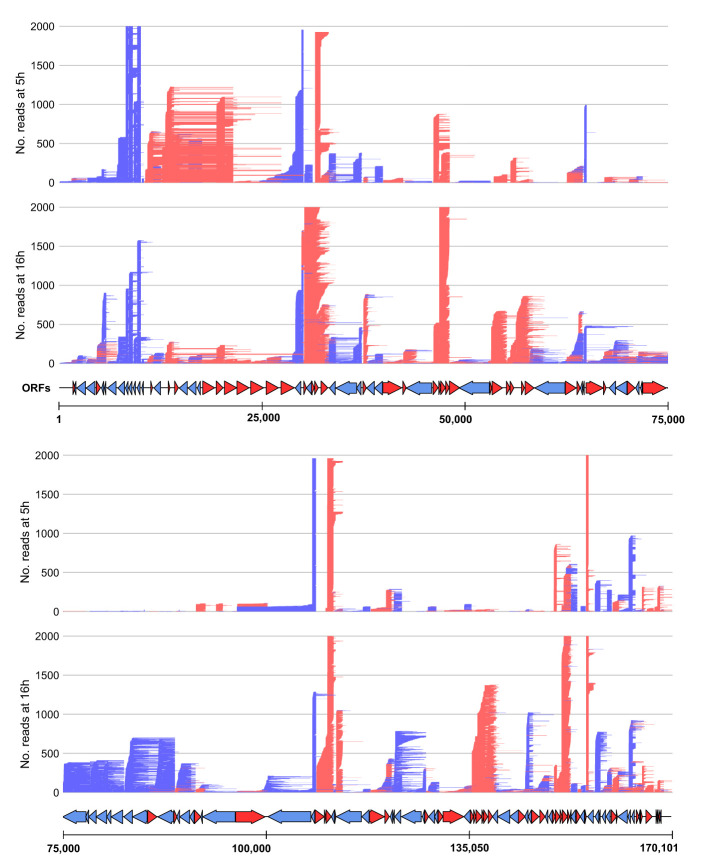
Mapping of the long-read sequencing (LRS) reads to the BA71V genome. Visualized in R using the gggenes (65) package. For visualization purposes, the genome was split in half, and each panel shows the long-read sequencing reads aligned from 5 hpi to 16 hpi (indicated by the left access). Arrows indicate BA71V ORFs oriented and colored according to their coding strand (red for plus, blue for minus).

**Figure 2:**
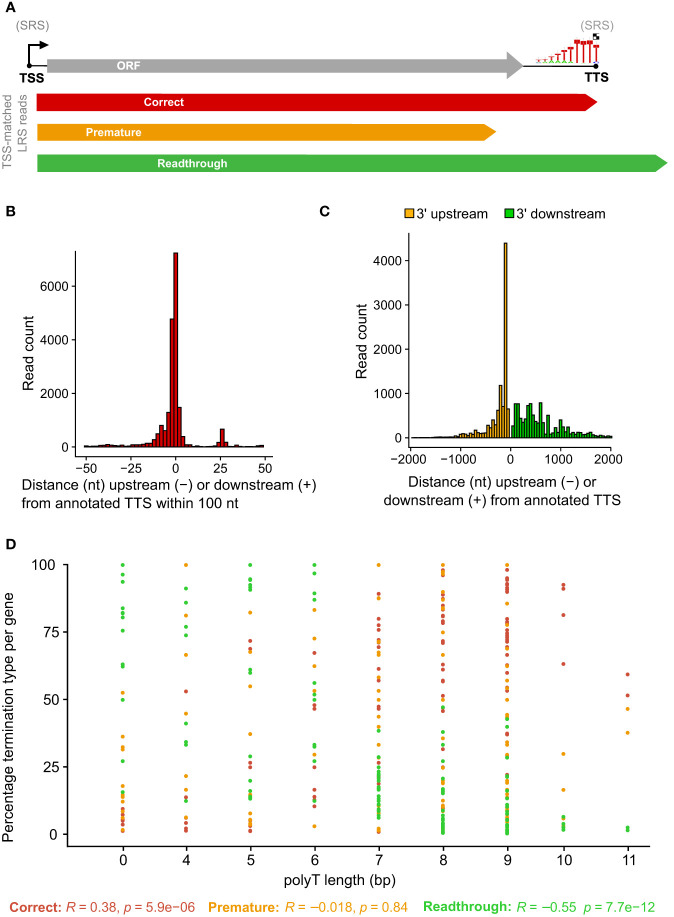
Comparison between LRS reads and SRS 3′ RNA-seq-based annotations. **(A)** Simplified schematic representation of how reads were classified according to their 3′ end location relative to the 3′ RNA-seq annotated pTTS. **(B)** Distribution of 3′ end (red) locations relative to that annotated 3′ RNA-seq TTS, from the 20,189 of 41,265 ONT reads which matched the 5′ end and the 3′ end—defined as within 100 bp of the TSS or TTS, respectively. **(C)** The locations of 3′ ends for the 21,076 reads which matched their 5′ end to the TSS location, but not the 3′ end with the pTTS location (bin width = 50 bp). **(D)** For each ASFV gene, the percentage of TSS-matched LRS reads that terminate prematurely, correctly, or readthrough relative to the SRS pTSS. Pearson correlation coefficients and *p*-values for percentage termination type versus polyT length at the SRS pTTS are shown underneath.

To probe for similarities and differences between the 5′ and 3′ ends of ASFV transcripts obtained by short- and long-read sequencing (SRS and LRS, respectively), we systematically compared the SRS techniques 5′ CAGE and 3′ RNA-seq, with LRS Oxford Nanopore Technologies (ONT) sequencing results. We carried out ASFV infection as described previously (4) and isolated total RNA at 5 and 16 h post-infection (hpi) representing early and late stages of infection. Libraries were prepared and sequenced according to the manufacturer’s instructions for ONT native RNA sequencing. We first mapped LRS reads to the BA71V genome (**Figure 1**) and compared the 5′ and 3′ termini to the SRS-derived previously annotated TSSs and transcription termination sites (TTSs), respectively (4). This allowed us to follow each transcript from its originating TSS to its 3′ end, as well as allowed us to analyze where this occurred relative to a gene’s ORF and SRS-annotated TTS. We defined each transcript as either terminating prematurely (Pre), correctly (Corr), or reading through (RT) relative to the SRS-annotated TTS (**Figure 2A**). Similar to previous observations using ONT native RNA-seq, the mRNA 5′ ends derived from LRS were not well-resolved relative to the TSS annotated by SRS CAGE-seq (61, 62). In contrast, the 3′ mRNA ends derived from LRS showed a close proximity with TTS mapped by 3′ RNA-seq SRS, typically within 10 nt (**Figure 2B**). This outcome is overall consistent with native RNA sequencing where the 3′ end resolution is better than the 5′ end resolution (66). One of our aims was to create a connection between specific promoter and terminator utilization. Due to the variable 5′ read end locations, we chose a window of within a hundred base pairs of the CAGE-seq TSS to qualify as a cognate or “matched” 5′ end.

This article is focused on RNA 3′ end formation which can be the result of transcription termination and/or processing; we apply a technique (native RNA-seq) that is optimized to isolate and sequence the 3′-polyadenylated transcripts. Unlike 3′ RNA-seq, native RNA-seq is not subject to the transcript-internal mispriming at A-rich sequences as it specifically selects RNA 3′ ends to initiate the sequencing. However, it does require transcripts to be polyadenylated as previously reported for ASFV mRNAs (2, 4). Of the 41,265 reads originating at a mapped ASFV promoter, the 3′ ends from approximately half the mRNAs (20,189 reads) were matched to that same gene’s primary (p)TTS (from 151 genes in total) mapped by 3′ RNA-seq SRS (**Figure 2B**). The pTTS was previously defined as the RNA 3′ with the largest number of reads downstream of that gene’s ORF. The other half of the LRS reads terminated >100 nt either upstream or downstream of the SRS-defined pTTS (**Figure 2C**); these 3′ termini are potentially generated by premature termination, RNA processing, or terminator readthrough, respectively. The biological importance of terminator readthrough relates to the possibility of multicistronic mRNAs (61, 62). However, it is not certain if the additional ORFs downstream of the first ORF will be translated in the infected cell, especially as no internal ribosome binding sites have been identified in ASFV. The fact that approximately half of the 3′ termini are not associated with primary transcription terminators suggests a complex termination landscape, suggesting multiple transcription termination and additional RNA 3′ formation processes.

### Detailed comparison of LRS TTS mapping with 3′ RNA-seq

A total of 10,885 LRS reads, from 151 BA71V (Vero-adapted ASFV strain) genes (4), matched both their LRS 5′ and 3′ ends to SRS pTSS and pTTS, respectively, corroborating our LRS approach. What makes a terminator strong, i.e., associated with low readthrough? From previous results, the SRS pTTSs were associated with either a polyT motif or no motif (4). This primary sequence motif consists of >4 T residues in the coding DNA strand, corresponding to >4 U residues in the mRNA. The number of U residues correlated with the proportion of correctly terminating transcripts (*R* = 0.38) and anticorrelated with the proportion of mRNA 3′ ends generated by terminator readthrough (*R* = −0.55), while we found no correlation with premature termination (*R* = −0.018) (**Figure 2D**). In essence, longer T stretches reduce terminator readthrough. Given that ASFV is AT-rich (39% GC content in BA71V), it is common to find polyT and polyA sequences. There are 3,743 ≥4 T stretches across both strands of the BA71V genome, which may necessitate a means of control beyond the sequence context alone, e.g., by termination factors.

### Motifs enriched at the 3′ ends of ASFV mRNAs

**Figure 3:**
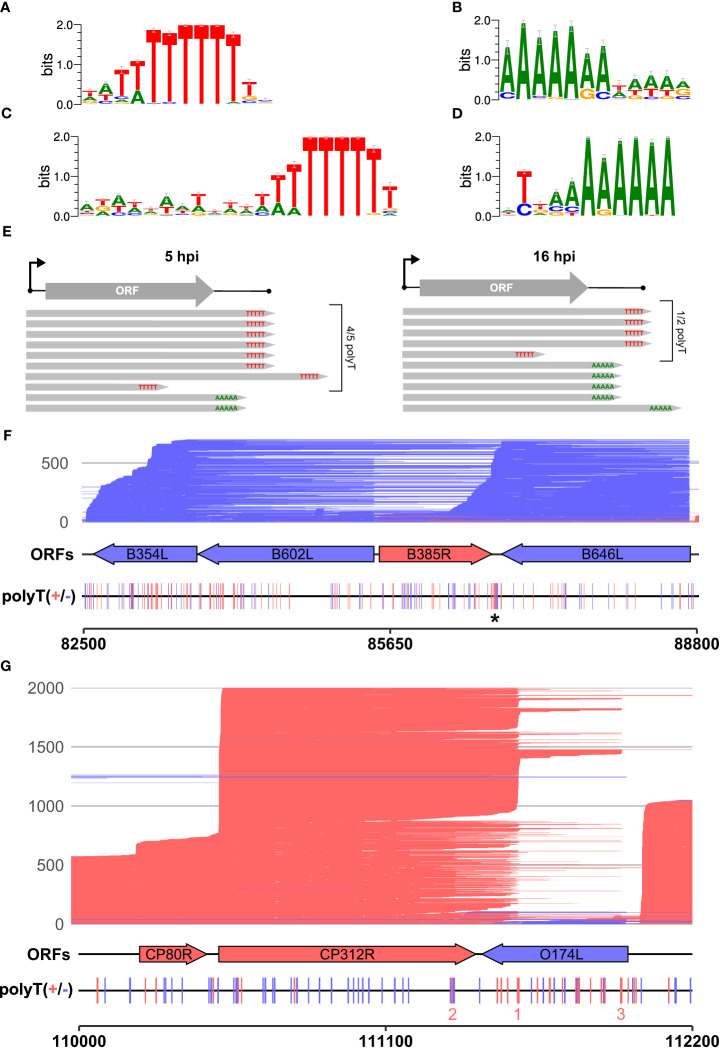
The 3′ ends of reads: enriched motifs and gene examples. MEME motif searches were carried out on all reads whose 5′ ends matched the CAGE-seq data for an annotated gene. The 3′ end nt of each read plus and minus 20 nt on either side were extracted for 3,823 and 9,216 total reads from 5 h to 16 h, respectively. **(A, B)** The first and second most significant motifs, respectively, detected from reads at 5 h. **(A)** This was found at 958 sites (E-value 3.2e−188) and **(B)** at 177 sites (E-value 1.1e-028). **(C, D)** The first (685 sites, E-value: 8.3e−050) and second motifs (580 sites, E-value 2.4e−005) found at the 3′ ends of reads from 16 h, respectively. **(E)** Summary schematic of analysis and results from panels **(A–D)**, i.e., after matching reads from 5 hpi to 16 hpi to their respective TSSs; the significant motifs found at 3′ ends of reads were mostly polyT (polyU) during early infection and an almost equal mix of polyT and polyA during late infection. **(F)** Full-length transcript landscape including, and downstream of, the gene *B646L*, representing non-discrete termination sites. The asterisk (*) indicates a polyT-rich region that could facilitate termination for either of the genes *B385R* and *B646L* but shows no clear enrichment of 3′ ends. **(G)** Full-length transcript landscape surrounding the gene *CP312R*, representing discrete termination sites. Reads are capped at 2,000 total reads for visualization. Total reads from 16 h are shown for the region of the BA71V genome indicated with the bottom scale for both **(F, G)**. Blue (minus) and red (plus) indicate strandedness of ORFs, polyT stretches of ≥4, and reads.

We scrutinized the genome-wide enrichment of any motifs at the RNA 3′ ends during early (**Figures 3A, B**) and late infection (**Figures 3C, D**). We observed a clear enrichment of polyT motifs at 3′ ends during both early and late infection, while the second most common motif was a polyA. The polyT motif frequency was fivefold that of polyA at 5 hpi, decreasing to parity at 16 hpi (summarized in **Figure 3E**). This is consistent with our observations based on SRS 3′ RNA-seq, which identified polyT terminators as more prevalent among early compared with late genes. In our previous SRS 3′ RNA-seq approach, polyA signatures were filtered out due to the possibility of transcript-internal mispriming (4), but this independent verification by LRS native RNA-seq (free of any primed PCR step) demonstrates that there are indeed genome-templated polyA sequences at the 3′ ends of ASFV mRNAs.

Since a polyA stretch on one DNA strand corresponds to a polyT stretch on the other, it is possible that the head-on collision of transcription elongation complexes (TECs) on convergent gene pairs results in the termination of either or both RNAPs on such a site (67). The two genes *B646L* and *B385R* provide an example of a converging gene pair (**Figure 3F**). *B646L* is a well-characterized and highly expressed late gene that encodes the capsid protein p72. While its TSS determined by CAGE-seq was distinct and clear, the TTS signal was scattered, with multiple associated SRS 3′ RNA-seq peaks located downstream of its stop codon. LRS demonstrates that B646L transcription starts consistently at the *B646L* promoter with little or no readthrough originating from upstream genes B117L or B407L (**Figure 3F**). The *B646L* transcript 3′ ends, however, are located over a broad region downstream of its stop codon. There are 21 polyT stretches (≥4 T’s) in the downstream region of *B646L*, with enrichment of polyT stretches on both strands in the intergenic region with the converging *B385R* gene (**Figure 3F**, asterisk). However, rather than terminating transcription consistently at any of these clear terminator motifs, many *B646L* transcripts consistently read through into the downstream region of *B385R*. Given that the converging *B385R* gene is expressed at much lower levels, it is tempting to speculate that the high expression levels of *B646L* are connected to this poor termination behavior. For example, multiple TECs in tandem could “force” RNAP through termination signals and rarely collide with a TEC transcribing the *B385R* gene.

The pattern observed with readthrough from *B646L* is not a universal rule, however, as can be observed with the highly expressed early gene *CP312R* that converges with the less well-expressed *O174L* (**Figure 3G**). *CP312R* mRNAs are initiated by the *CP312R* promoter, or alternatively by the utilization of the upstream *CP80R* and *CP530R* promoters, due to readthrough. Regardless of the promoter utilization, *CP312R*, *CP80R*, and *CP530R* transcripts consistently terminate at discrete sites: one pTTS (1 in **Figure 3G**) and two secondary termination sites (2 and 3 in **Figure 3G**). Given these contrasting patterns for genes with seemingly similar local organization, it raises the question of how much a gene’s local environment affects its termination patterns, and if this differs between early and late ASFV genes. Further comparison between converging gene pairs with more similar gene expression levels shows reads stopping just short of one another at the two pTTS in **Supplementary Figure 1A** or overlapping and generating a “clash region” of ~100 bp between *A276R* and *A238L* in **Supplementary Figure 1B**. This latter gene pair also shows an example of the same terminator being used by two genes, but on opposing strands: the pTTS of *A276R* and an npTTS of *A238L* are located on the same terminator, but on opposing strands, generating a polyT and polyA motif, respectively.

### The role of gene organization for transcription termination

**Figure 4:**
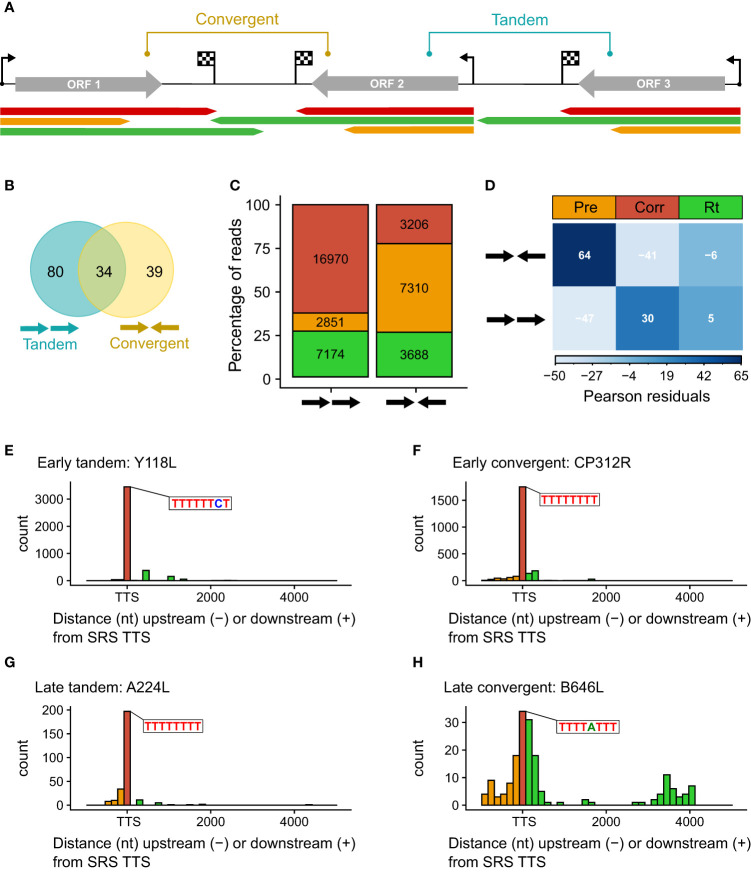
**(A)** Schematic representing the categories of tandem and convergent gene layout, along with examples of LRS read mapping, colored according to termination type. **(B)** Summary Venn diagram representing the positioning of genes relative to one another across the BA71V genome. BEDTools was used to classify each of the 153 BA71V genes [as annotated in Cackett et al. (4)], according to the next genes up- and downstream. **(C)** Bar chart showing the proportion of reads showing each termination type versus their gene organization (tandem or convergent). Termination type is colored as before (red, amber, and green represent correct, premature, and readthrough, respectively). The bar height represents the percentage of termination type per gene layout type, annotated with the number of reads per termination type. These reads were extracted from the 41,265 which matched the 5′ ends from CAGE-seq; 24 reads were excluded due to no annotated gene downstream (at the genome termini). **(D)** Correlation matrix plot following a chi-squared test of independence on read frequency per termination type against each gene layout. Pearson’s chi-squared test of independence: *χ* ^2^ = 4214.7, *p*-value < 0.001. The scale indicates Pearson residuals, with navy indicating a strong positive association (e.g., between converging genes and reads prematurely terminating or between contiguous genes and readthrough) and white indicating a strong negative association (e.g., between converging genes and reads prematurely reading through or between contiguous genes and reads prematurely terminating). **(E–H)** Distribution of distances between the 3′ read ends from LRS versus the 3′ RNA-seq TTSs. Shown as histograms with a bin width of 150 nt for every graph. There are two examples each for early tandem and convergent genes (*Y118L* and *CP312R*) and late tandem and convergent genes (*A224L* and *B646L*). Color scheme as before: amber, red, and green represent premature, correct, and readthrough termination relative to the 3′ RNA-seq TTS (or ORF stop codon in the case of A104R, shown in blue).

The ~170-kb ASFV BA71V genome is densely packed with genes on both strands: 73 on the plus strand and 80 on the minus strand in the genome U18466.2. Gene pairs can be oriented in convergent (head-to-head) or tandem (contiguous genes) arrangement (as illustrated in **Figure 4A**), but one gene can be assigned to both categories when it is in tandem relative to the upstream gene and convergent to the downstream gene, or vice versa (**Figure 4B**). To systematically assess any dependence on the orientation of the downstream gene, we only considered genes according to their relative orientation to the closest downstream gene. Genes were classified as tandem or converging as summarized in **Table 2** (detailed in **Supplementary Table 1**). Overall, ASFV genes are distributed equally on either strand and tend to be non-overlapping, with some exceptions (4). Our analysis shows that tandem gene pairs are more prominent than convergent ones genome-wide and that there are less convergent early compared with late genes (~38% and ~51%, respectively). A key factor in this is the layout of predominantly tandem-oriented multigene family (MGF) members toward the genome termini. The evolutionary selection pressures that have resulted in this genome organization are not fully understood but may have been shaped by optimizing or facilitating correct gene expression levels including transcription termination.

**Table 2:** Comparison between the BA71V genes classified as “late” or “early” according to their differential expression between 5 h and 16 h from CAGE-seq, as well as each gene’s relationship to the next gene downstream.

Gene type	Relationship to the downstream gene	Number of genes	Proportion of genes per gene type (%)
Early	⇨⇨ (tandem)	37	61.7
Early	⇨⇦ (converging)	23	38.3
Late	⇨⇨ (tandem)	41	49.4
Late	⇨⇦ (converging)	42	50.6
NC	⇨⇨ (tandem)	1	16.7
NC	⇨⇦ (converging)	5	83.3

There were a total of 149 genes for which we had CAGE-seq data and had a gene downstream, and a detailed list of these is shown in **Supplementary Table 1**.

Arrows represent relative gene orientation of each gene to its closest neighbor downstream, with head-to-head arrows illustrating converging genes (on opposing strands), and head-to-tail arrows representing genes in tandem orientation (on the same strand).

We compared the proportion of mRNAs associated with i) correct, ii) premature termination, or iii) terminator readthrough for the tandem or convergent gene orientation (**Figure 4C**). The results show that premature termination is dominant among convergent genes (**Figure 4D**). **Figures 4E–H** illustrate the quantification of 3′ ends from four genes with different expression and genomic context patterns: early tandem Y118L (**Figure 4E**), early convergent *CP312R* (**Figure 4F**), late tandem *A224L* (**Figure 4G**), and late convergent *B646L* (**Figure 4H**). These results indicate that both early genes and the late tandem gene *A224L* have relatively consistent 3′ end formation patterns, albeit the latter with more premature termination. In contrast, late convergent *B646L* is characterized by an abundance of both premature and readthrough transcripts (see also **Figure 3F**).

### *De-novo* definition of TTSs using LRS

Thus far, we have considered each full LRS-mapped transcript solely in the context of termination sites defined using SRS, but only approximately half of the LRS reads matched the previously mapped pTTS. Our analysis suggests that SRS had correctly picked up an accumulation of 3′ ends for these genes and, therefore, a putative termination site (previously defined as a pTTS). However, many of the reads originating from these gene promoters were not terminating at the SRS TTS. **Supplementary Figure 2** shows examples of early and late genes whose mRNA 3′ ends were predominantly not located at the SRS-annotated pTTS.

**Figure 5:**
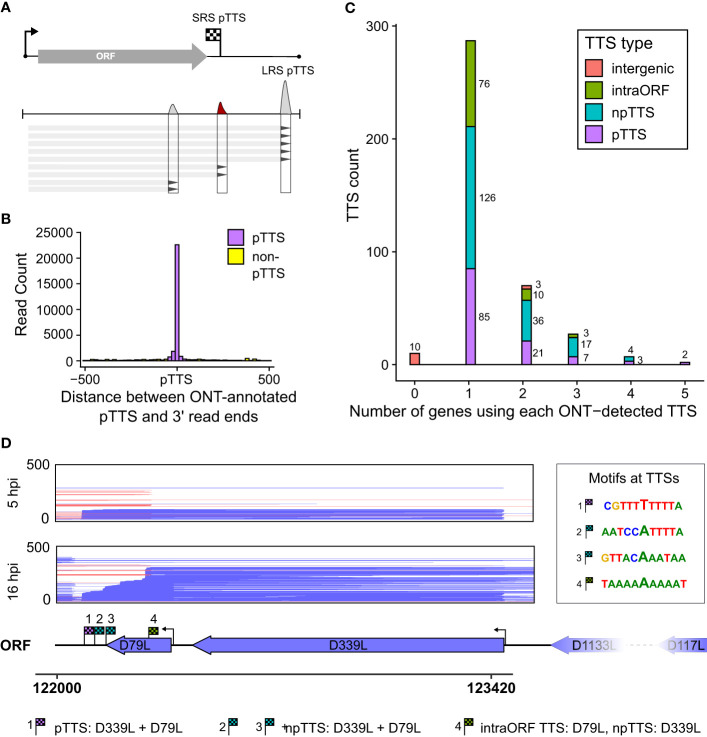
LRS TTSs versus LRS 3′ ends. **(A)** Summary schematic for how locations with an accumulation of 3′ transcript ends were extracted from LRS reads and used for peak calling to identify LRS TTSs. **(B)** For all the 41,265 reads whose 5′ ends matched the CAGE-seq TSSs, the distribution of 3′ end locations is shown relative to the newly LRS-defined pTTS—colored magenta if within 50 bp of this pTTS, all other TTSs in green. **(C)** For the total 376 LRS-defined TTSs, their location and role relative to the gene from which the transcripts originated and its ORF were defined into four groups: pTTS for the most-used TTS downstream of a gene’s ORF, npTTS for less used TTSs downstream of a gene, intra-ORF for TTSs within the originating gene’s ORF, and intergenic if the transcripts terminating at a TTS had no matching 5′ end to an annotated gene. **(D)** An example of TTS sharing between genes *D79L* and *D339L*, showing reads aligned in this region capped at 500 reads for visualization purposes. TTSs for both genes are annotated and their surrounding motifs are shown on the right.

The power of LRS is to capture the mRNA transcript in its entirety and to unequivocally assign which promoters (or TSSs) are associated with which gene terminators (or TTSs). We identified locations with an accumulation of RNA 3′ ends, newly defining these LRS TTSs (**Figure 5A**). For each of these 376 LRS TTSs, we searched upstream to identify cognate TSSs. We detected TTSs originating from 115 gene TSSs in total and subsequently defined the strongest TTS downstream of any ORF within that transcription unit as the pTTS (TTS, from here onwards refers to LRS TTS). For the >40,000 reads whose 5′ ends matched a TSS, the vast majority of their 3′ ends coincide with the new LRS-defined pTTSs (**Figure 5B**), indicating LRS TTSs captured the 3′ end landscape in a more comprehensive fashion, compared with those defined via SRS (as illustrated in **Supplementary Figure 3A** versus **Supplementary Figure 3B**). **Supplementary Table 2** shows matched LRS and SRS TTSs, demonstrating 80 being perfectly matched, i.e., both methods defined them as either primary or non-primary and correctly identified them from which gene promoter transcripts they originated.

### ASFV genes commonly share and utilize multiple termination sites

**Figure 6:**
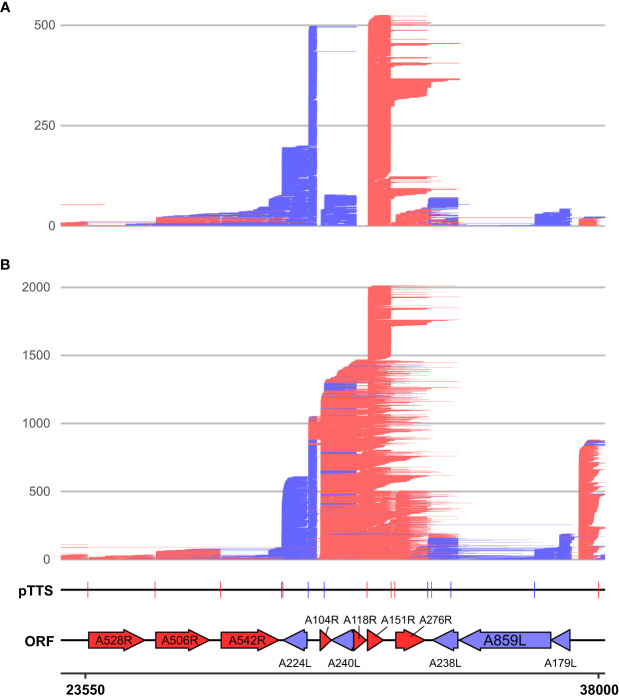
LRS reads aligning between 23,550 and 38,000 on the BA71V genome at **(A)** 5 hpi and **(B)** 16 hpi. Novel LRS-annotated pTTSs and known ORFs are labeled, while all strands, ORFs, and TTSs are colored red or blue according to the strand (plus and minus, respectively). *A528R*, *A506R*, and *A542R* are also known as MGF505-7R, MGF505-8R, and MGF505-10R, respectively.

We categorized the novel 376 sites into four different “TTS types” (illustrated in **Supplementary Figure 3B**) representing the location of each TTS with respect to their originating gene, or lack thereof in the case of “intergenic” TTSs (**Figure 5C**; **Supplementary Table 2**). TTSs were defined as primary or non-primary TTSs (pTTS or npTTS) according to their prevalence (number of reads). TTSs within the gene’s ORF were classified as “intra-ORF.” This approach clarified and highlighted that many TTSs did indeed originate from a single TSS. However, many TTSs (primary and non-primary) were being used by several genes. The sharing of pTTS and npTTS is schematically illustrated in **Supplementary Figure 3C**, and two example genes (*D79L* and *D339L*) that share both primary and non-primary termination sites are shown in **Figure 5D**. We furthermore found that early genes and highly expressed genes on average use a greater number of TTSs although we cannot rule out that the detection limit of the method contributes to this effect (**Supplementary Figures 3D, E**, respectively). Three genes (*A151R*, *A224L*, and *A104R*) annotated in **Supplementary Figure 3D** had an unusually large number of TTSs and were all found in close proximity to one another (**Figure 6**). This region shows high levels of readthrough but consistent usage of distinct TTSs by both early and late transcripts (**Figures 6A, B**, respectively). Genes in this region are examples of both extensive sharing of the same TTSs, as well as genes that use a high number of them, such as *A104R* whose transcripts extend kilobases beyond its stop codon, utilizing the same terminators of *A118R*, *A151R*, and *A276R*. *A104R* is a highly expressed late gene, and analysis of the 3′ end formation between *A104R* and similarly expressed *B646L*, *K78R*, and *E184L* shows high levels of heterogeneity, with premature and readthrough transcripts being highly abundant (**Supplementary Figure 4**).

### LRS termination site motifs and tandem terminators

**Figure 7:**
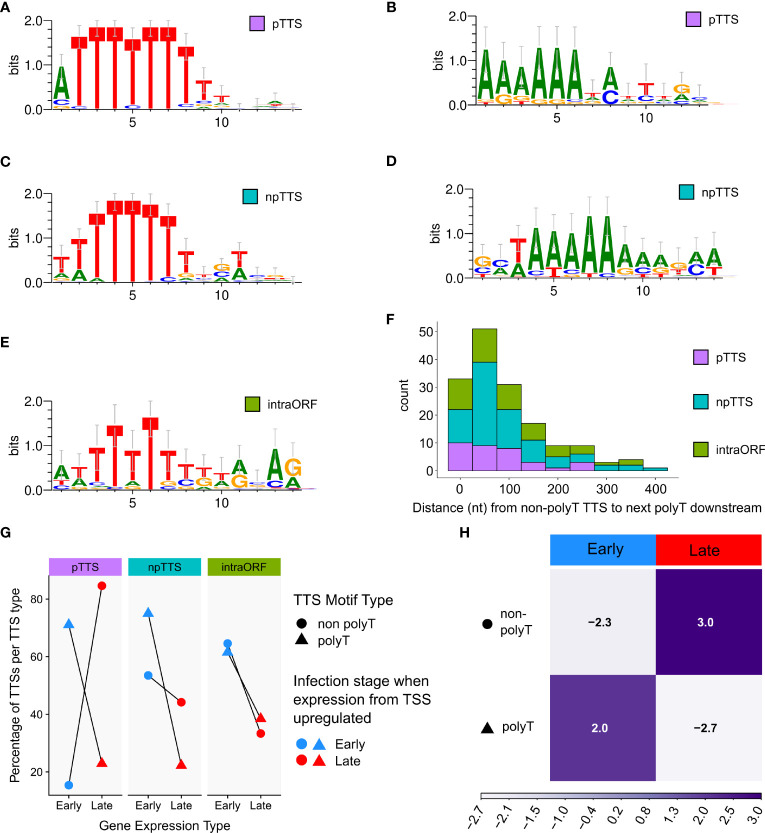
Significantly enriched DNA motifs detected via MEME, searching the 10 bp up- and downstream of the TTS, separated according to type (pTTS, npTTS, and intra-ORF), ordered according to abundance. **(A, B)** The only two significant motifs detected at 71 (E-values 3.2e−056) and 21 (3.5e−007) sites, respectively, from a total of 111 pTTSs. **(C)** The most significant motif detected from 179 npTTSs, which was found in 65 sites (E-value 8.9e−036). **(D)** The second most common motif detected among npTTSs was detected at 27 sites (E-value 9.9e−005). **(E)** This was the only significant motif found at 22 of the 87 intra-ORF TTSs (E-value 2.4e−002). WebLogo was used to create these motifs from the MEME fasta output. **(F)** The distances in nt from each of the 158 lacking any polyT TTSs to the next polyT downstream. One non-polyT TTS was omitted as it had no polyT downstream—being at the genome terminus. **(G)** A summary of TTS types according to their classification as primary, non-primary, or intra-ORF, whether their sequence contains a polyT or not and if the TSS from which their reads predominantly originate was defined as an early or late gene TSS according to previous CAGE-seq data. **(H)** Correlation matrix plot following a chi-squared test of independence, on the number of early and late gene terminators per motif category. Pearson’s chi-squared test of independence: *χ* ^2^ = 24.9, *p*-value < 0.001. The scale indicates Pearson residuals, with dark purple indicating a positive association and white indicating a negative association.

After defining TTSs based on LRS data, we investigated the sequence motifs at the different TTS types including 111 pTTSs (**Figures 7A, B**), 179 npTTSs (**Figures 7C, D**), and 87 intra-ORF-TTSs (**Figure 7E**). **Supplementary Table 2** lists these TTSs and their main gene users, matches them to their SRS counterparts, and describes their motifs, while **Supplementary Table 3** lists their location in bed format. In good agreement with the SRS results, the most common pTTS, npTTS, and intra-ORF terminator signature was a polyT tract (**Figures 7A, C, E**). The second most common motif of pTTSs and npTTSs was a polyA tract (**Figures 7B, D**, respectively). Furthermore, we found no significant evidence of secondary structure formation to be more likely in the 50 nt of RNA upstream of the TTSs detected, after calculating their minimal folding energies (MFEs) and comparing them to the genomic background (**Supplementary Figure 5**). For the TTSs lacking discernable terminator sequence motifs, we examined the sequence downstream and found that the majority of non-polyT TTSs were within 100 bp of a putative polyT terminator sequence further downstream. Based on our results, we cannot rule out that transcription terminated at these downstream polyT motifs and that the observed mRNA 3′ ends were generated by cleavage or trimming, i.e., the outcome of co- or posttranscriptional endo- or exonucleolytic events. Lastly, we compared the different TTS types with the differential gene expression of their associated mRNAs. In good agreement with our previously published CAGE-seq data, early genes had a higher proportion of polyT terminators compared with late genes (**Figure 7G**), while TTSs with a polyA or non-discernable motifs were more commonly associated with late genes (**Figure 7H**). However, the most significant and discernable motif was still the polyT across TTS types.

### The ASFV core RNAP is able to recognize polyT terminators independently of termination factors

Our genome-wide analysis of transcription termination sites highlighted the importance of polyT signature motifs in ASFV. While the host RNAPII depends on termination and polyadenylation factors (68), archaeal RNAP (10, 69) and RNAPIII (12, 63, 64) are able to faithfully terminate transcription at polyT motifs, without the requirement for upstream RNA secondary structures that characterize canonical bacterial intrinsic terminators abundant in bacteria (11, 70). VACV RNAP transcription termination depends on factors, some of which are conserved between VACV and ASFV (**Table 1**), despite the ASFV enzyme being structurally closer to its host RNAPII counterpart (19). To probe whether the ASFV RNAP conforms to the factor-dependent paradigm of RNAPII and VACV RNAP or is more like intrinsic termination found in RNAPIII and archaeal RNAP, we tested whether a recombinant ASFV RNAP made of the eight core subunits was able to recognize a range of terminators identified in our sequencing data. We recently reported the production of catalytically active, wholly recombinant ASFV RNAP expressed in insect cells that is suitable for a rigorous functional analysis *in vitro* (19).

**Figure 8:**
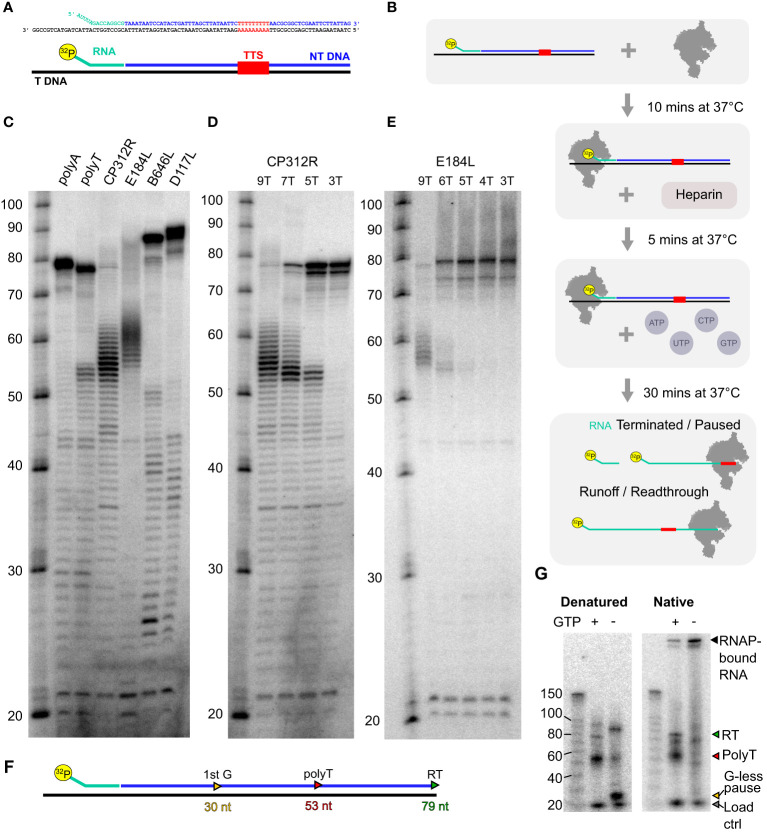
*In-vitro* transcription termination with recombinant core ASFV-RNAP. **(A)** Example of scaffold (native CP312R) with TTS motif identified from transcriptomic analysis. **(B)** Schematic of the step-by-step process for carrying out transcription elongation assay. The main final products being the ^32^P-labeled RNA which had not been elongation, products of pausing or termination at terminators, and finally readthrough transcripts which are generated from RNAP reaching the end of the template strand. **(C)** Following the process in **(B)**, denatured samples were run on an 11% TBE-polyacrylamide 7 M urea denaturing sequencing gel for a range of scaffolds. The sequences of template and non-template strands, as well as the lengths of transcribed products, are shown in **Supplementary Table 5**. The polyA and polyT transcripts were synthetic based on a previous work (69), while *CP312R* (polyT), *CP530R* (no motif), and *D117L* (polyA) were native ASFV terminators. **(D)** Transcripts from native *CP312R* polyT motif (9 nt), followed by *CP312R* 7T, 5T, and 3T as the same scaffolds with subsequent replacement of 2T with 2A in the sequence (see **Supplementary Table 5**). **(E)** Transcripts from native *E184L* polyT motif (9 nt), followed by *E184L* 6T, 5T, 4T, and 3T, as the same scaffolds with subsequent replacement of a T with an A in the sequence. **(F)** Schematic summary of how the *CP312R* scaffold sequence generates transcripts with specific lengths in the presence and absence of GTP in transcription reactions. **(G)** *In-vitro* reactions from *CP312R* 7T in **(C)** ran on a TGX 4%–15% gel under native conditions in TG buffer. Lanes where GTP was omitted from the reactions are indicated, inducing a pausing prior to the terminator motif, wherein the sequence contains only 2 G’s.

Based on a protocol we previously developed for archaeal RNAP (69), we assembled transcription elongation complexes (TECs) with ASFV RNAP and a nucleic acid scaffold consisting of an RNA primer, template, and non-template DNA strand (**Figure 8A**). Following preincubation to allow for TEC assembly, we challenged the reaction with heparin to reduce primer-independent transcription. In the presence of NTP substrates, RNAP will extend the ^32^P-labeled RNA primer and carry out transcription elongation independent of promoter sequences or transcription initiation factors (**Figure 8B**). The reaction products are separated on denaturing polyacrylamide (“sequencing”) gels to characterize RNA products at single-nucleotide resolution or on native gels to probe for the association/dissociation of the RNA from the TEC (69). We designed a range of templates encoding terminators mapped using SRS and LRS methods including those associated with the genes *CP312R* (polyT), *E184L* (polyT), *D117L* (polyA), and *B646L* (polyA). In addition, we included synthetic templates used in the archaeal termination study containing embedded 7T or 7A motifs as positive and negative controls, respectively. The corresponding read alignments for the sequences used *in vitro* are shown in **Supplementary Figure 5** (*CP312R* and *E184L*) and **Supplementary Figure 6** (*D117L* and *B646L*). As is the case with the archaeal RNAP, the synthetic polyT template resulted in termination along with runoff transcripts, and the polyA template exclusively produced the latter. All terminator constructs generated multiple bands, suggesting that the mRNA 3′ formation of RNAP *in vitro* can accommodate a degree of flexibility. Factor-independent (i.e., intrinsic) transcription termination is frequently accompanied by the formation of several termination products in the closely related RNAPs of archaea (71) and eukaryotic RNAPIII (12, 63, 64). In comparison, intrinsic termination by bacterial RNAP tends to be more precise, likely due its dependence on RNA hairpin secondary structures that are not found in ASFV (**Supplementary Figure 5**) (4, 72). Alternatively, VACV-like (24) promoter-proximal RNAP slippage in ASFV could generate transcripts with varying lengths (4). The ASFV *CP312R* and *E184L* terminators, each having a stretch of nine T residues, turned out to be very efficient terminators *in vitro* without significant transcription readthrough. In stark contrast, the ASFV *D117L* and *B646L* terminators associated with polyA motifs did not lead to termination *in vitro* but only produced runoff transcripts (**Figure 8C**). To test the correlation between the number of T residues and termination efficiency, we utilized mutant variants of the CP312R terminator varying the number of T’s from 9 to 7, 5, and 3 (**Figure 8D**). Our results show a dose–response-dependent decrease in terminated transcripts and a concomitant increase in terminator readthrough RNA when decreasing the number of T residues. Almost no readthrough was observed with nine T’s, and no polyT-dependent termination could be observed with three T’s. To ascertain that this phenomenon is not restricted to the *CP312R* terminator, we introduced similar variations in T content in the terminator of the *E184L* gene (**Figure 8E**). The results were directly comparable to *CP312R*, cementing the view that the number of T residues determines the termination efficiency of ASFV RNAP.

This indicates that the core RNAP alone is capable of terminating transcription, without any added termination factors, on polyT motif of ≥5 nt in length. Denaturing gels as in **Figures 8C–E** cannot discriminate between paused TECs and the outcome of *bona fide* termination, where the RNA has dissociated from the TEC. To distinguish pausing from termination, we separated released RNA from TEC by native gel electrophoresis. Both polyT-associated (red triangle) and runoff transcripts (green triangle) are present in native and denaturing conditions showing that the RNA has been released from the TEC during termination. We cannot rule out that a small fraction of RNA was dissociated subsequent to the transcription reaction during electrophoresis. To rule out this background signal, we included a control using a paused TEC generated by nucleotide limitation. The strong *CP312R* scaffold encodes a 30-nt transcript (**Figure 8F**) in the absence of GTP (**Figure 8G**, detailed layout in **Supplementary Figure 8A**). This stable paused TEC forms a band with low mobility on native gels, which demonstrates that the majority of the RNA remains associated with RNAP in paused complexes, with only a minor proportion of RNA released (“−GTP” in **Figure 8G**). Without nucleotide restriction (“+GTP”), the signal of the retained complex decreases significantly as they terminate transcription and release the RNA. The same pattern occurred throughout all ASFV scaffolds used (**Supplementary Figures 7B, C**).

In summary, the ASFV core RNAP can terminate transcription at polyT motifs independently of transcription termination factors, and the exact mRNA 3′ end shows some flexibility. The polyA signals associated with some late gene terminators *in vivo* cannot enable termination without additional factors *in vitro*.

## Discussion

In the current study, we have applied LRS to analyze genome-wide termination in ASFV and compared it to our previous 3′ end mapping using SRS 3′ RNA-seq (4). Both studies indicated a clear-cut relationship between early genes and the use of the polyT terminator motif. Late transcription termination is also associated with polyT terminators, but also A-rich motifs, while 3′ end formation appears more variable. The mechanism by which ASFV uses the polyT terminator motif generating a polyU tract at the 3′ ends of transcripts appears to share similarities to bacteria (11), archaea (10, 69), and RNAPIII (12, 63, 64). In contrast to bacterial intrinsic terminators that are reliant on an RNA hairpin upstream of the polyU (11), we have found no evidence of stem-loop formation associated with ASFV terminators. While a polyT signal is necessary and sufficient to terminate transcription in archaea, the termination factor archaeal cleavage and polyadenylation specificity factor 1 (aCPSF1) that is homologous to the RNAPII termination factors CPSF73 assists termination in a fashion that is enhanced by the recognition of RNA polyU stretches by the KH domains of aCPSF1, which are upstream of the termination site (8, 73, 74).

Our *in-vitro* transcription assays demonstrate that the ASFV core RNAP is able to terminate transcription without the strict requirement of predicted termination factors (**Table 1**). A run of five consecutive T’s (or U’s in the RNA sequence) is sufficient for ASFV RNAP to stop transcription elongation, while the longer the motif length, the stronger the stop signal, with nine T’s being sufficient to abolish transcription readthrough. Furthermore, we see evidence that the RNAP also releases transcripts following polyT terminators, suggesting ASFV RNAP is capable of intrinsic termination activity in response to this signal (**Figure 8**)—akin to intrinsic transcription termination in archaea (10, 69). Of course, as in archaea where polyT readthrough is also common (75), this intrinsic activity does not exclude the possibility that ASFV carries out factor-dependent transcription termination. The polyA terminator sequences were not able to terminate ASFV core RNAP *in vitro*, but it may also be the case that these sites are generated by other means, such as processing by RNases, subsequent to termination. ASFV may utilize a similar termination mechanism to archaea, whereby RNAP can intrinsically terminate at a polyT, but termination factors enhance the process in a polyT signal-dependent fashion (8, 73, 74). ASFV encodes multiple predicted transcription termination factors, though is not clear whether these enhance transcript release in an ATP-dependent manner, akin to the VACV system (see below), following pausing at a polyT terminator.

The utilization of termination factors in VACV differs between early, intermediate, and late gene transcription. During intermediate and late VACV infection, termination is thought to be facilitated by the H5 factor aka VLTF-4 (76, 77), reminiscent of activities by CPSF and the cleavage stimulatory factor (CstF), in their recognition of a polyadenylation signal (PAS) and promoting cleavage (78–80). VACV G2 and the DNA helicase A18 interact with H5 *in vivo* (81), where A18 facilitates transcript release in an ATP-dependent fashion (36, 82). Interestingly, H5 and G2 have also been reported to enhance elongation (30, 31), suggesting that the interplay between H5, G2, and A18 can shift the balance between elongation and termination. However, the mechanism and structural basis of termination by these factors remains opaque. Termination of early VACV genes is not reliant on A18, but by D11 (aka NPH-I) (83, 84) or I8 (NPH-II) (85–87), which terminate transcription immediately following pyrimidine-rich sequences, both in the absence and presence of the upstream UUUUUNU motif (23).

Like VACV, ASFV encodes NPH-I (*Q706L*) and NPH-II (*B962L*) homologs, both of which are found in viral particles suggesting their role during early transcription (3), similar to VACV (85). Recent structural studies suggest that VACV NPH-I facilitates promoter escape by an unusual upstream “DNA scrunching” mechanism (88). Importantly, the complete VACV–RNAP complex (including NPH-I) is capable of site-specific transcription termination (20, 89). In ASFV, no homolog for H5 nor G2 has been identified, though there are two ASFV homologs for the intermediate and late termination factors VACV-A18:*QP509L* and *A859L* (32–35, 90). ASFV-QP509L is the best candidate for an A18 homolog as both are ~500 residues in length and closely related at the sequence level (EMBOSS Needle (91) sequence identity: 19.1%). ASFV-A859L encodes a larger product that only partially aligns to residues 45–793 with VACV-A18, according to similar pairwise alignment (sequence identity: 14.0%). Transcriptome analyses show that *QP509L*, *A859L*, and *Q706L* genes are all upregulated during late ASFV infection. Only VACV-A18 is present in VACV particles indicating a function for early gene transcription (92), while its ASFV homologs QP509L and A859L are not found in ASFV particles (3). Neither *QP509L* nor *A859L* is essential for the virus as either could be deleted individually in ASFV (93, 94); as the double knockout was not prepared, it is possible that QP509L and A859L are functionally redundant. *Q706L* and *QP509L* are both expressed in mid to late infection (95), but only Q706L is packaged in viral particles (3). Intriguingly, their knockdown hindered ASFV replication and disrupted late transcription, but did not affect early gene expression (96). Future work would perhaps benefit from LRS following infection with the A18 homolog deletion strains or putative termination factor knockdowns described above, to assess how the absence of these factors affects termination readthrough. It would be expected that the absence of *bona fide* transcription termination factors would lead to an increase in readthrough, while knocking out elongation factors should have the opposing effect. Similar experiments in VACV involved mutations made to factors like A18 (82), NPH-I (D11L) (97), NPH-II, or I8 (98) and provided vital evidence for their predicted roles in transcription termination.

LRS was previously used by Olasz and colleagues (61, 62) to investigate RNA extracted from ASFV-infected porcine macrophages. The study included a comparison of LRS to SRS data obtained by traditional RNA-seq and the analysis of ASFV transcript isoforms with their 5′ and 3′ ends (62). Their results confirmed the commonly reported diversity in viral transcript lengths [as summarized previously (90)] and importantly showed that long transcripts included multiple consecutive ORFs. The relatively low sequencing depth of this study limited its reach, and the pooling of samples precluded an analysis of differential gene expression or varying read lengths throughout the ASFV infection time course. As temporal gene expression is key to understanding ASFV biology, we sought to fill this knowledge gap with the current study, which demonstrates significant changes in the 3′ end landscape between early and late infection.

One of the remaining key questions is how the non-polyT TTSs we detected factor into the ASFV transcriptomic landscape. The 3′ RNA-seq initially failed to identify many late gene TTSs, compared with those from early genes. LRS indicated that late gene transcript 3′ ends are indeed enriched at polyT motifs, but also at a polyA (**Figure 7**), which would have been filtered out from our 3′ RNA-seq data to remove potential mispriming (4). RNA 3′ end enrichment at polyA motifs is clearly more common during late infection and for late-classified genes (**Figure 7G**), though the reason for this remains enigmatic. Due to the AT-rich nature of the ASFV genome, polyT sequences are widespread, ensuring most of the non-polyT TTSs we detected were close to polyT sequences downstream (**Figure 7F**). Future work should investigate how 3′ end formation occurs among those non-canonical non-polyT TTSs, which become more frequent during late infection. It will be also important to investigate whether they are indeed *bona fide* termination sites or arising through some other mechanism such as 3′ end processing. Transcription terminator readthrough of convergent genes can produce mRNA 3′ regions that are complementary to each other and thus form dsRNAs, which in turn can trigger antiviral responses (99, 100) including the interferon response, RNA interference by Dicer (dsRNA targeting) (101, 102), and activation of RNase L (ssRNA cleavage) (103). The latter acts in concert with oligoadenylate synthetase gene 1 to inhibit ASFV replication (104).

**Figure 9:**
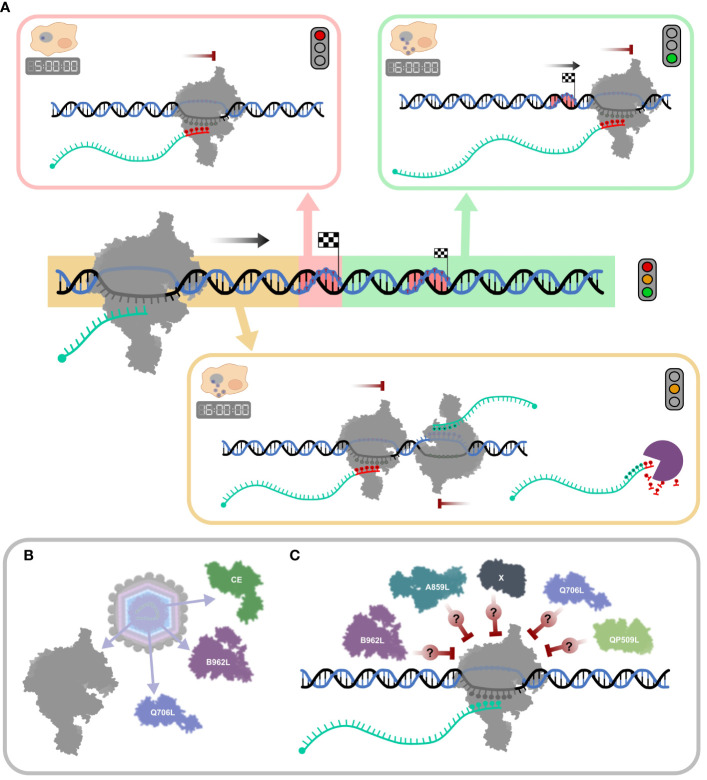
Schematic summary of ASFV transcription termination and putative mechanisms of RNA 3′ end formation. **(A)** The mechanisms for “correct” termination (red highlight), premature termination (yellow highlight), and terminator readthrough (green highlight) are illustrated in boxes. During the early stages of infection (5 hpi), termination is dominated by concise mRNA 3′ formation associated with strong polyU stretches at RNA 3′ ends (red nt). A more complex mRNA 3′ end landscape can be observed in late infection (16 hpi), alongside concise termination, abundant terminator readthrough (green), and premature 3′ end formation (yellow), many of which are not associated with polyT motifs. Transcripts appearing as prematurely terminated include mRNA 3′ ends generated by *bona fide* termination, head-on collisions of RNAPs which transcribe convergent gene pairs, or alternatively by mRNA degradation or processing. **(B)** ASFV particles include at least two termination factor candidates, Q706L and B962L, and the CE, important for termination in VACV (described in **Table 1**). **(C)** The ASFV genome encodes additional putative termination factors including A859L and QP509L, but their molecular mechanisms and exact roles during termination are still not well understood (96).

While the role and extent of RNA 3′ processing of ASFV mRNAs remains unclear, it is possible and even likely that some of the mapped RNA 3′ ends are not “nascent” termination products, but generated by processing, and this applies particularly to late infection. **Figure 9A** summarizes the broad patterns of transcription termination in ASFV. While this accounts for intrinsic factor-independent termination as demonstrated *in vitro* (**Figure 8**), ASFV encodes several termination factor candidates (**Table 1**) that contribute to the formation of the viral transcription termination landscape. Those found in ASFV particles (3) are likely utilized during the early stages of infection (**Figure 9B**). However, their mechanisms of action remain poorly understood. Likewise, the role of other putative termination factors or as-yet undiscovered candidates remains opaque (**Figure 9C**).

A vital tool in the production of attenuated ASFV strains is a detailed gene expression landscape, playing a key role in vaccine development. Compromising ASFV pathogenicity, attenuating the virus can be achieved by many different means, and the molecular machineries responsible for viral gene expression are key targets genes in this context—as well as for the screening and development of specific inhibitors with the potential to act as antiviral drugs.

## Summary

- The transcription termination landscape of ASFV is highly complex.
- Termination sites can be associated with either polyT or polyA DNA motifs or not be associated with any sequence signatures.
- PolyT terminators of different lengths are the dominant termination signals, with a larger number of T residues resulting in more efficient termination and less transcription readthrough.
- PolyT motifs become less common during late infection.
- ASFV polyT terminators are not associated with RNA hairpin secondary structures.
- PolyT terminators are recognized directly by the eight-subunit ASFV core RNAP independently of termination factors.
- ASFV polyT terminator architecture is evolutionary conserved with archaeal RNAP and eukaryotic RNAPIII systems.
- mRNA 3′ complexity increases greatly during late infection due to variations in TTS usage and possibly due to increased RNA processing and degradation.
- Transcription readthrough is a prominent feature of ASFV terminators and results in polycistronic mRNAs, although it remains uncertain to which extent downstream ORFs are translated.
- The molecular mechanisms of non-polyT-facilitated termination are unknown but may involve termination factors, head-on collisions with RNAP transcription elongation complexes (TECs) on convergent genes, collisions with replication forks, and other DNA-binding factors such as chromatin proteins (e.g., A104R).

## Future directions

The recombinant ASFV RNAP and *in-vitro* transcription assays will now empower us to characterize the molecular mechanisms of transcription termination in ASFV under rigorous conditions. The combination of functional genomics studies, like the one reported here, and *in-vitro* experiments will enable a comprehensive yet detailed understanding of transcription termination in ASFV. The ensuing burning research questions include the following:

- What is the structure and function of the predicted ASFV termination factors (**Table 1**)?
- Which genes are terminated by which termination factors during early and late infection?
- How does the depletion of termination factors alter the mRNA 3′ patterns *in vivo*? Does 3′ end formation differ between ASFV strains?
- How are ASFV termination factors and their mechanisms related to other NCLDV viruses and to cellular RNAP transcription systems?
- Does chromatinization by DNA-binding factors modulate transcription initiation and termination of ASFV genes?
- Are downstream ORFs in multicistronic mRNAs translated in ASFV-infected cells?

## Materials and methods

### Long-read sequencing of ASFV-BA71V: Oxford Nanopore MinION library preparation and sequencing

A total of four RNA samples were extracted under the same conditions as for previous BA71V transcriptomic work (4): two replicates from 5 h and 16 h post-infection. RNA sequencing libraries were prepared with the Direct RNA Sequencing Kit (Oxford Nanopore or “ONT,” SQK-RNA002) according to the manufacturer’s instructions. In brief, any polyadenylated RNA present was annealed to an oligo-dT primer along with an adapter to facilitate reverse transcription (RT) from native 3′ ends of transcripts with SuperScript IV (Thermo Fisher, Waltham, USA). Magnetic beads (Agencourt AMPure XP, Beckman Coulter, Brea, USA) were mixed with 1 µl of RNasin Ribonuclease Inhibitor (Promega, Madison, USA) per 100 µl beads. Following RT, the sample was mixed with this bead–inhibitor mix, and using a magnetic rack, the beads were cleaned with 70% EtOH. After elution from the beads in nuclease-free water, a sequencing adapter was ligated to the RNA–DNA hybrid, bound to fresh magnetic beads, and washed with the kit’s wash buffer and magnetic rack, before eluting in its elution buffer. Samples were prepared for loading onto a MinION flow cell (FLO-MINSP6) according to the manufacturer’s instructions. Samples were sequenced until there were no remaining pores in the flow cell (1 flow cell per sample, taking up to 72 h), and the results were output in FAST5 format.

### Basecalling and mapping of nanopore sequencing reads

Guppy (v4.4.2, Oxford Nanopore) was used for basecalling, i.e., converting the FAST5 voltage signal files into FASTQ sequencing data files (parameters: –flowcell FLO-MIN106 –kit SQK-RNA002 –trim_strategy none –fast5_out –reverse_sequence on –calib_detect on -r). Output FASTQ files were then concatenated to generate FASTQ files for each sample, containing both Vero host and ASFV-BA71V reads. Minimap2 (105) was used to map reads from FASTQ files to either the ASFV-BA71V (U18466.2) or Vero (GCF_000409795.2 *Chlorocebus sabaeus* 1.1) genomes, after generating index.mmi files for each genome to improve mapping speed. Mapping command: minimap2 -ax splice -uf -d -a [genome file].mmi [sample file].fastq > [sample file].sam. For visualization, SAM files were converted to BAM files using Samtools (106), which was also used to extract lengths of reads mapping to each genome.

### Genome-wide comparison to LRS 5′ and 3′ ends to 5′ CAGE-seq and 3′ RNA-seq

BAM files were sorted and indexed using IGV Tools (107) before BEDTools (108) was used to return LRS reads which overlapped our newly annotated BA71V genome in GFF3 format [from Cackett et al. (4)], using the command: bedtools intersect -wo -s -a [.gff3 file] -b [.bam file]. Each resulting table was imported into RStudio (109) (Version 1.1.456, R version: 3.6.3) in which most subsequent analysis and data visualization took place, predominantly using the packages dplyr (110) and ggplot2 (111). At this stage, results from replicates were pooled into two groups: ASFV reads overlapping genes at 5 h and reads overlapping genes at 16 h. For each read that overlapped an annotated gene on the same strand, these were filtered according to firstly whether the 5′ end of the read was within 100 nt of the CAGE-seq-annotated TSS. Then, reads were filtered according to where the 3′ ends of reads were located, relative to the 3′ RNA-seq-annotated pTTS (4): at the pTTS (classed as “correct” termination), upstream of the pTTS (“premature”), and downstream of the pTTS (“readthrough”). For all the reads which mapped close to the 5′ ends, all reads would be assigned to either of these three categories, with their frequencies per gene being compared between early and late genes (assigned from CAGE-seq), between time points overall, or between timepoints when the pTTS contained a polyT sequence motif, and according to the polyT length (number of consecutive T’s). All this was saved as a data.frame in RStudio for downstream analysis.

### Relationship between polyT presence and termination types at TTSs

Genome-wide polyT occurrences were identified via searching for any “TTTT” motif across each strand of the ASFV-BA71V genome using IGV Tools “Find Motif” function, and polyT locations were exported in BED format. BED files were then sorted (command: sort -k1,1 -k2,2n [.bed] > [sorted.bed]). Sorted BED file coordinates were merged with BEDTools, to combine consecutive stretches of >4 T’s into a single annotation in the BED file for each strand (command: bedtools merge -i [sorted.bed] > [merge.bed]). BAM alignments were converted to BW format via deepTools (112), with one file for each strand (command: bamCoverage –bam [.bam file] –outFileName [.bw file] –outFileFormat bigwig –binSize 1 –filterRNAstrand [forward/reverse] –normalizeUsing CPM).

### Visualization of aligned LRS reads

Alignments shown in **Figures 1**, **3**, **5**, **6**, as well as in **Supplementary Figures 1, 6, 7**, were all generated with R in RStudio after deepTools (112) was used to separately pool the BA71V-aligned reads for the 5-h and 16-h time points and convert them from BAM to BED format before conversion to GFF. Each of these GFF files was imported into RStudio using import.gff3() from the package rtracklayer (113). Annotations such as TTS or polyT locations were imported in six-column BED format using import.bed(). Alignment figures were generated via the packages ggplot, ggbio, and rtracklayer, and the gggenes package (65) was used to generate the gene maps beneath each alignment.

### Genome-wide transcription termination patterns

The layout of ASFV genes initially summarized in **Figure 4** was manually annotated for the 153 ASFV-BA71V genes, according to each gene’s relationship to its closest neighboring genes. Genes whose neighbors shared the same strand and therefore direction were classed as “contiguous,” “clashing” if its 3′ end was directed toward the 5′ end of its neighbor, and “diverging” if two genes’ 5′ ends began together and were directed away from one another on opposing strands. Due to the compact nature of the BA71V genome and some genes overlapping, these categorizations were not mutually exclusive. Genes were defined as only either clashing or contiguous (diverging genes could be either), only according to the direction of the gene downstream. For reads that matched their 5′ ends within 100 nt of TSSs of annotated genes, a BED file only containing the last nt at the 3′ end was extracted. BEDTools slop was used to expand the 3′ end nt location of each read plus and minus 20 nt on either side. BEDTools getfasta was then used to extract the genomic sequences within these regions. Fasta files were filtered for duplicate sequences using sRNAtoolbox (114) to reduce bias from highly expressed genes—with many identical terminator motifs. The filtered sequences were then searched for enriched motifs using the MEME Suite (115) (searching for three motifs 5–20 nt in length, in zoops mode).

### Defining TTSs from LRS and enriched predicting motifs

To annotate TTSs *de novo* using the LRS data, each sample coverage BAM file was first converted into a BW file via deepTools (112) with only coverage for the last 3′ end nt of each read. TTS prediction was carried out using these BW files as input, using the CAGEfightR (116) package in R, as carried out for our 3′ RNA-seq TTS prediction (4). The 376 TTSs found via CAGEfightR for peak calling were annotated according to their position relative to the closest ORF using BEDTools closest -s and manually, if applicable. There were four different “TTS types”: firstly, the vast majority of TTSs were defined as primary or non-primary TTSs (pTTS or npTTS), pTTS meaning that the highest number of reads originating from a particular gene’s TSS would have their 3′ ends located at this specific site. Secondly, if there were further enriched sites used by genes, which were utilized less than its pTTS, these were defined as npTTSs. The designation of pTTS or npTTS was done via calculating the percentage of each gene’s reads (5′ ends matching) terminating at a particular TTS (3′ ends within 100 nt) and manually checked from alignments in IGV. Thirdly, if after matching to a gene’s TSS, the 3′ end was found within that gene’s ORF, this was described as “intra-ORF.” Lastly, for reads whose 5′ ends did not match to any annotated gene, these were called “intergenic.” These different annotations also confirmed that often TTSs could be used by multiple genes—defined as reads whose 3′ ends were within 100 nt of the same TTS but whose 5′ ends were within 100 nt of TSSs for different genes.

After annotating the LRS TTSs with their originating TSSs and defining each as pTTS (111 TTSs), 179 npTTS, or 87 intra-ORF-TTS, these TTS sequences were searched for enriched motifs. This was carried out as described above, except with the region 10 bp up- and downstream of each TTS, rather than the 3′ ends of the reads. The MEME suite was also similarly used to find three motifs for each TTS type (in zoops mode, 5–21 nt in length). All motifs were made using the sequence output from MEME, listing all the sequences contributing to each motif, converted to.fasta format, and input into WebLogo 3 (117) (www.weblogo.threeplusone.com/create.cgi). According to the appearance of each TTS motif, they were defined as a “polyT” or “non_polyT.” These TTS types were compared in RStudio using mainly dplyr (110) to the expression of the main gene user of each TTS, i.e., which gene matched its TSS to the highest proportion of 5′ read ends, whose 3′ ends then matched to that TTS. The location of each LRS TTS along with details of its matched gene and expression and their surrounding sequences are listed in **Supplementary Table 2**. **Supplementary Table 3** contains a summary of all LRS TTS locations across the BA71V genome, including in BED file format, named according to the most common gene users of each site.

### Prediction of minimal folding energy

RNAfold (118) was used to predict RNA minimal folding energy of 50 nt upstream of all the annotated TTSs including the TTS position. BEDTools random was used to extract 10,000 genomic sequences of the same length to serve as a background. The values in kcal/mol are listed in **Supplementary Table 4**.

### Scaffolds for *in-vitro* transcription elongation assays

The RNA (RNA14 from Hirtreiter et al. (69), sequence: AUUUAGACCAGGCG) was ordered from GenScript, Piscataway, USA, and 10 µM of RNA14 was ^32^P-labeled with [γ-^32^P] ATP (Hartmann Analytic, Braunschweig, Germany), 1 µl of PNK, and 5 µl of PNK buffer (M0201S, NEB, Ipswich, USA), with 0.5 µl of RNasin Plus Ribonuclease Inhibitor (Promega, Madison, USA) before making up the volume to 50 µl with RNase/DNase-free H_2_O. After incubation for 1.5 h at 37°C, free [γ-32P] ATP was removed from the reactions via MicroSpin G-25 desalting columns (Cytiva, Marlborough, USA) as per the manufacturer’s instructions. The sequences for synthetic polyA and polyT template (T) sequences were from Hirtreiter et al. (69) Their corresponding non-template (NT) sequences were designed to anneal directly downstream of the annealed RNA, and this design was used for all RNA:dsDNA scaffolds. DNA oligos used for generating scaffolds used in transcription assays were ordered from Integrated DNA Technologies, Coralville, USA and Merck Life Science, Darmstadt, Germany (**Supplementary Table 5**). The DNA and RNA were annealed in a ratio of 10 µM:2.5 µM:2.5 µM of RNA:T:NT strands, respectively, in reaction volumes of 50 µl, with 1 µl of RNasin Plus Ribonuclease Inhibitor, 2 µl of 25× annealing buffer (250 mM of Tris–HCl pH 7.5, 1.25 M of NaCl), and the remaining volume made up with water. Annealing was carried out at 96°C for 1 min before 2 min at room temperature and then placed on ice before use in assays.

### *In-vitro* transcription elongation using recombinant ASFV-RNAP

Purified recombinant ASFV RNAP (60 nM) [produced as previously described (19)] was preincubated for 10 min at 37°C, with 0.5 µl of RNasin Plus Ribonuclease Inhibitor (Promega, Madison, USA) and 5.6 µl of the RNA:dsDNA scaffold prepared as above. Heparin solution was added to a final concentration of 1 µg/ml per reaction, followed by 5 min incubation at 37°C. Reactions were started by adding 2.6 µl of a master mix to generate final concentrations per reaction of 300 µM for each ATP, GTP, CTP, and UTP, as well as 25 mM of Tris–HCl pH 8, 3 mM of MgCl_2_, 50 mM of KCl, 7 mM of DTT, and 2.5 mg/ml of BSA. Reactions were then incubated for 30 min at 37°C and stopped either via loading directly onto a native gel (4%–20% TGX Bio-Rad, Hercules, USA) run in TG buffer or by the addition of denaturing Gel Loading Buffer II (Thermo Fisher Scientific) and boiling for 5 min at 95°C before loading onto a denaturing 11% TBE-polyacrylamide 7 M urea gel. The ladder used for polyacrylamide gel electrophoresis was the Decade Markers System (AM7778, Thermo Fisher Scientific).

## Data availability statement

The datasets presented in this study can be found in online repositories. The names of the repository/repositories and accession number(s) can be found below: https://www.ncbi.nlm.nih.gov/sra/, PRJNA1045388.

## Author contributions

GC: Conceptualization, Data curation, Formal analysis, Funding acquisition, Investigation, Methodology, Visualization, Writing – original draft, Writing – review & editing. MS: Methodology, Validation, Writing – review & editing. RP: Writing – review & editing, Methodology, Validation. CD: Writing – review & editing, Methodology. LD: Funding acquisition, Project administration, Supervision, Writing – review & editing. FW: Conceptualization, Funding acquisition, Project administration, Supervision, Writing – review & editing.
