# Supplementary material for: Transcription termination and readthrough in African swine fever virus
Source: Front Immunol. 2024 Mar 13;15:1350267. doi: 10.3389/fimmu.2024.1350267 (PMC10965686; doi:10.3389/fimmu.2024.1350267)
Supplement: Supplementary file 2 [file DataSheet_1.docx]

Supplementary Material

**Transcription Termination and Readthrough in African Swine Fever Virus**

**Gwenny Cackett *, Michal Sýkora, Raquel Portugal, Christopher Dulson, Linda Dixon and Finn Werner ***

*** Correspondence:** Gwenny Cackett g.cackett@ucl.ac.uk and Finn Werner f.werner@ucl.ac.uk

# Supplementary Table Legends

**Supplementary Table 1.** List of genes in BA71V genome (U18466.2) and their arrangements for relative to one another. Per gene definitions were decided according to each gene’s TTS position relative to the next genes downstream. Genes defined as ‘Converge’ were positioned head-to-head relative to the next gene downstream (and on the opposite strand) while genes classified as ‘Tandem’ were contiguous and on the same strand as the next gene downstream. In regions of the genome where genes are dense and overlapping, a single gene could often be both converging with one gene and tandem to another, these were classed as ‘Both’.

**Supplementary Table 2.** Details of all LRS TTSs detected using peak calling with CAGEfightR listed, including cluster names, main gene user of each TTS, each TTS genomic location, and the TTS type. The column ‘Other gene usage of TTS and converging gene pairs’ lists auxiliary users of the same TTS i.e. transcripts originating from other gene promoters whose 3’ ends end at this TTS. Additionally noted in this column, are cases where there was clear example of converging with specific genes (as Converge [gene name]) or non-coding antisense transcripts. Where applicable, the SRS TTS locations which matched the newly identified LRS TTSs (within 100 bp) are listed along with details of said SRS TTS. Columns under ‘SRS versus LRS annotated TTSs’ include agreement and disagreement between annotations obtained from both methods. The table also includes a summary of the expression profiles for the main gene users of each LRS TTS obtained from CAGEseq, and finally the sequence context surrounding the LRS TTS and whether a polyT was detected.

**Supplementary Table 3.** LRS TTS locations from Supplementary Table 2, included in bed file format.

**Supplementary Table 4.** Minimum Folding Energy (MFE) of 50 bp upstream of TTS or 10,000 random genomic locations (background).

**Supplementary Table 5.** List of RNA and DNA oligos used to generate scaffolds used for *in vitro* transcription reactions shown in Figure 8.

# Supplementary Figures


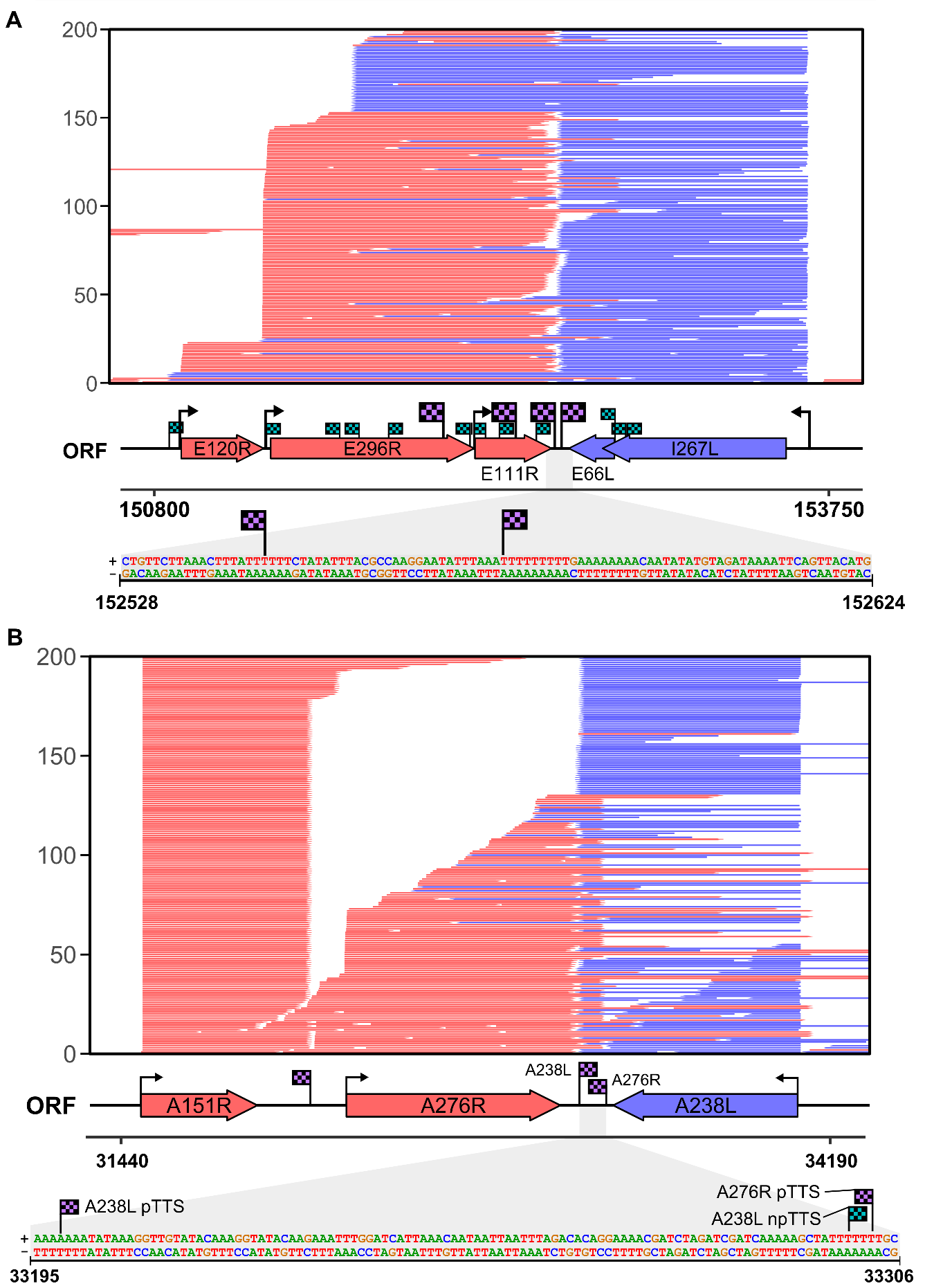


**Supplementary Figure 1.** Examples of read alignments in the regions surround convergent genes in ASFV. The reads shown are from 16 hpi. (A) The clashing read region of predominantly E296R and I267L. The purple flags indicate the pTTS for each gene, smaller blue flags indicate the large number of npTTSs across this region. (B) The clash read region of A276R and A238L Annotated as in (A) but only showing each pTTS.


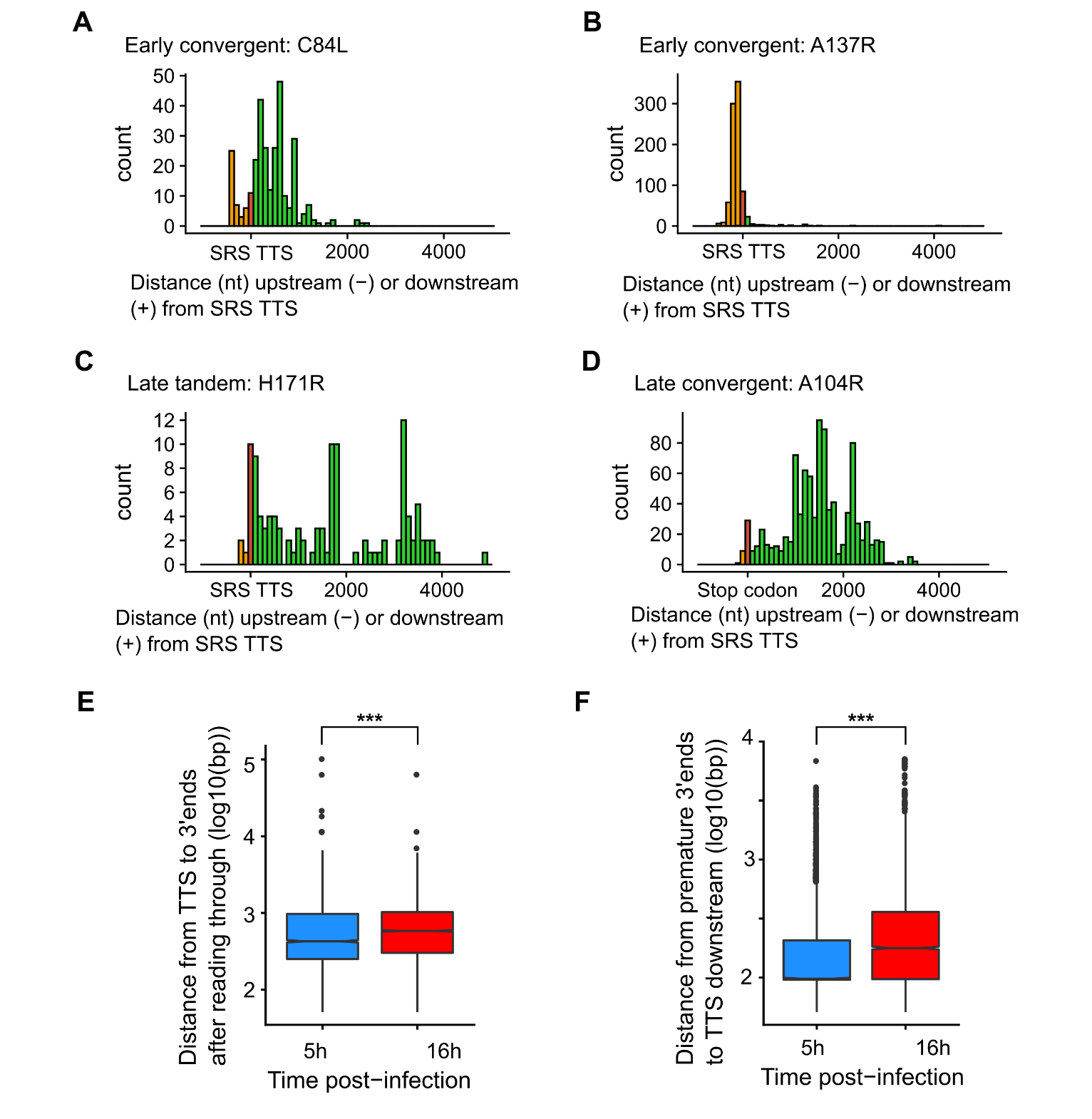


**Supplementary Figure 2.** Examples of genes whose LRS 3’ end distributions do not match predicted pTTS locations from SRS 3’ RNA-seq including early convergent genes C84L (A) and A137R (B). Late tandem gene H171R (C) whose SRS pTTS is the closest and highest point immediately downstream of the gene’s ORF, but the majority of 3’ ends are downstream of this SRS pTTS. (D) The wide distribution of 3’ ends from A104R transcripts, the majority extending over 1000 bp downstream of the ORF’s stop codon.


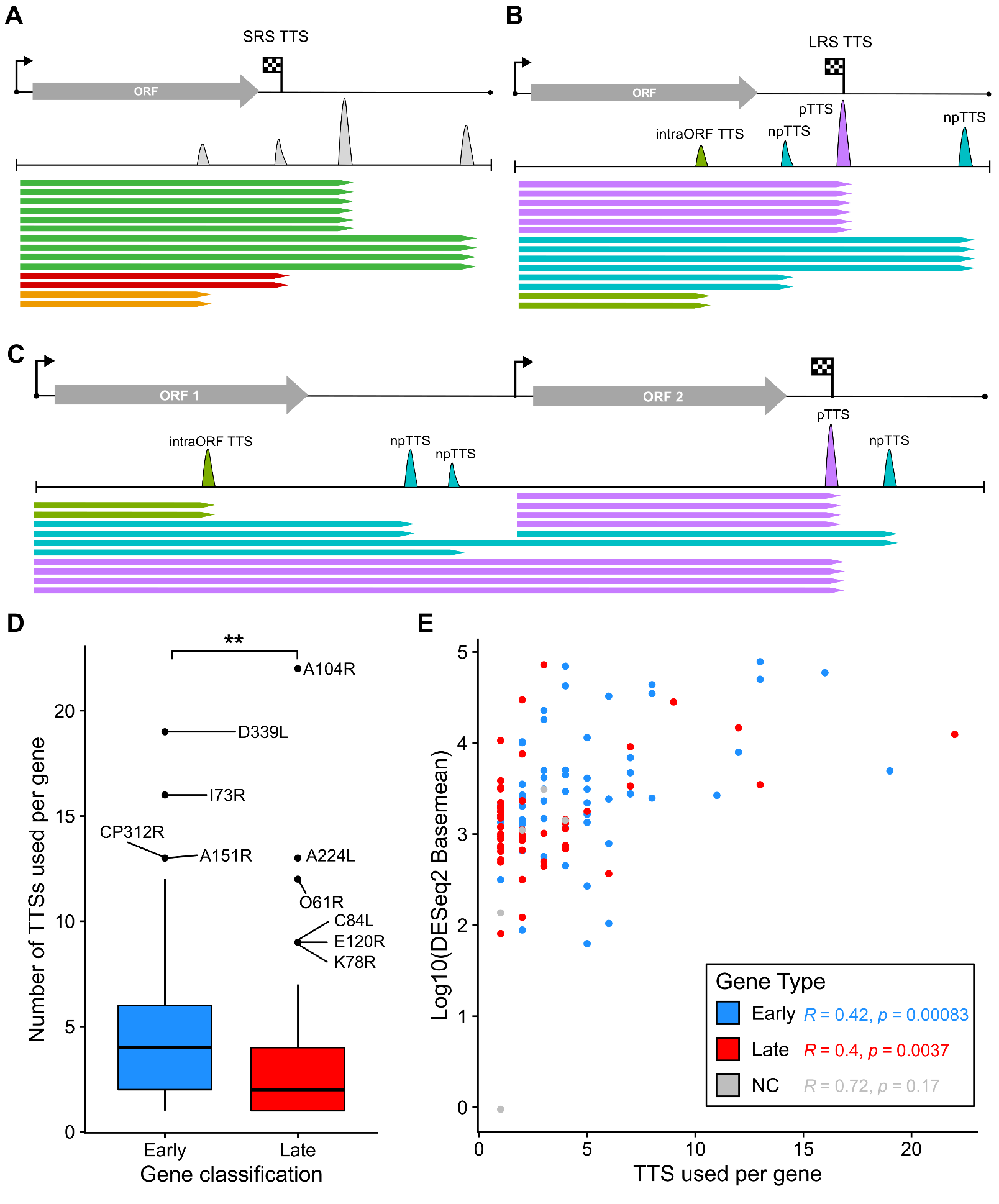


**Supplementary Figure 3.** Schematic representation of comparison between SRS and LRS datasets. (A) a common scenario when comparing SRS TTS annotations and LRS 3’ ends i.e. while there is an accumulation of 3’ ends in line with the SRS TTS location, the majority are further downstream. (B) Schematic of the new LRS-defined TTSs based on enrichment of 3’ LRS ends. (C) An example of how both a pTTS and npTTSs can be equally shared between 2 genes (e.g. D79L and D339L in Figure 5). (D) Comparison between the number of TTSs used per gene classified as early or late genes according to CAGE-seq. A total of 110 genes were compared, 59 were early genes and 51 were late genes. ** Indicates the significant different between the early and late gene TTS usage according to a Wilcoxon rank-sum test (p-value = 0.0004725). (E) Scatter plot between the number of TTSs used per gene and its log10(DESeq2 basemean expression), as defined in Cackett et al. 2020 ^1^. This comparison was across all 115 genes for which LRS TTSs were found and whose transcripts were quantified via CAGE-seq - the same 110 genes as in (D), plus the 5 non-classified (‘NC’) genes which were not found to be differentially expressed.


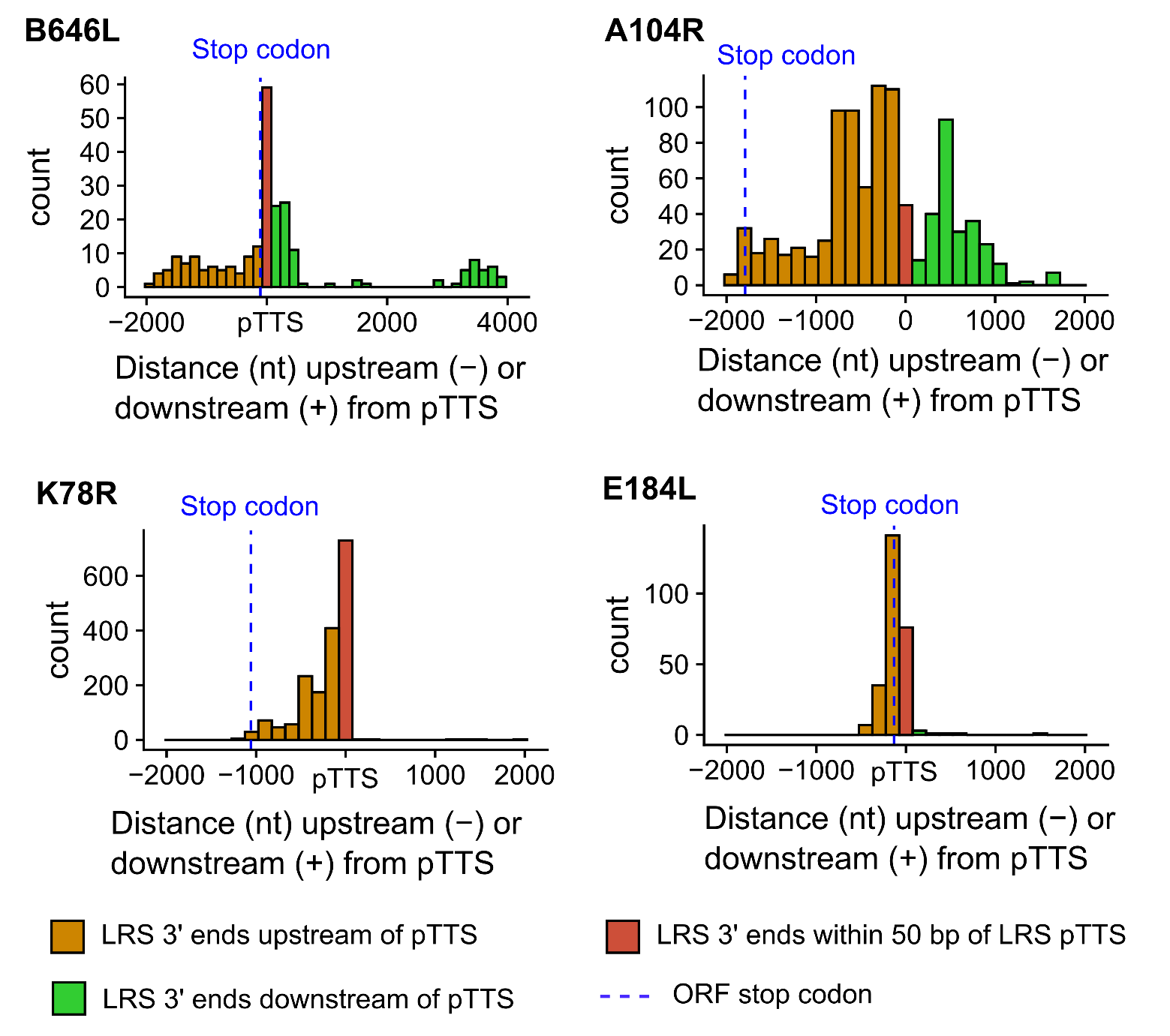


**Supplementary Figure 4. .** Examples of LRS 3’ end distributions for highly expressed late genes like B646L (top left). Bars of read 3’ end counts are coloured according to their position relative to each gene’s newly-annotated LRS pTTS. A blue dotted line indicated the end of the gene’s ORF.


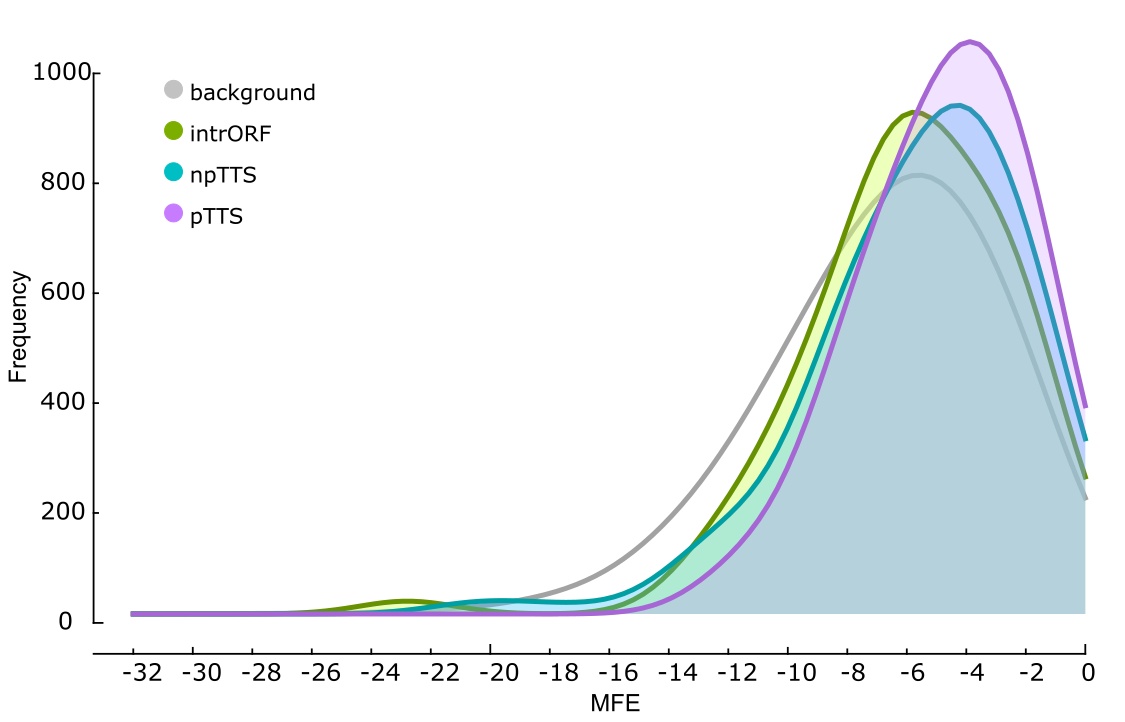


**Supplementary Figure 5.**

Density distributions of predicted minimal folding energies (MFE) of RNA structures (in kcal mol−1) for pTTS, npTTS, intraORF TTS and random genomic positions (background).


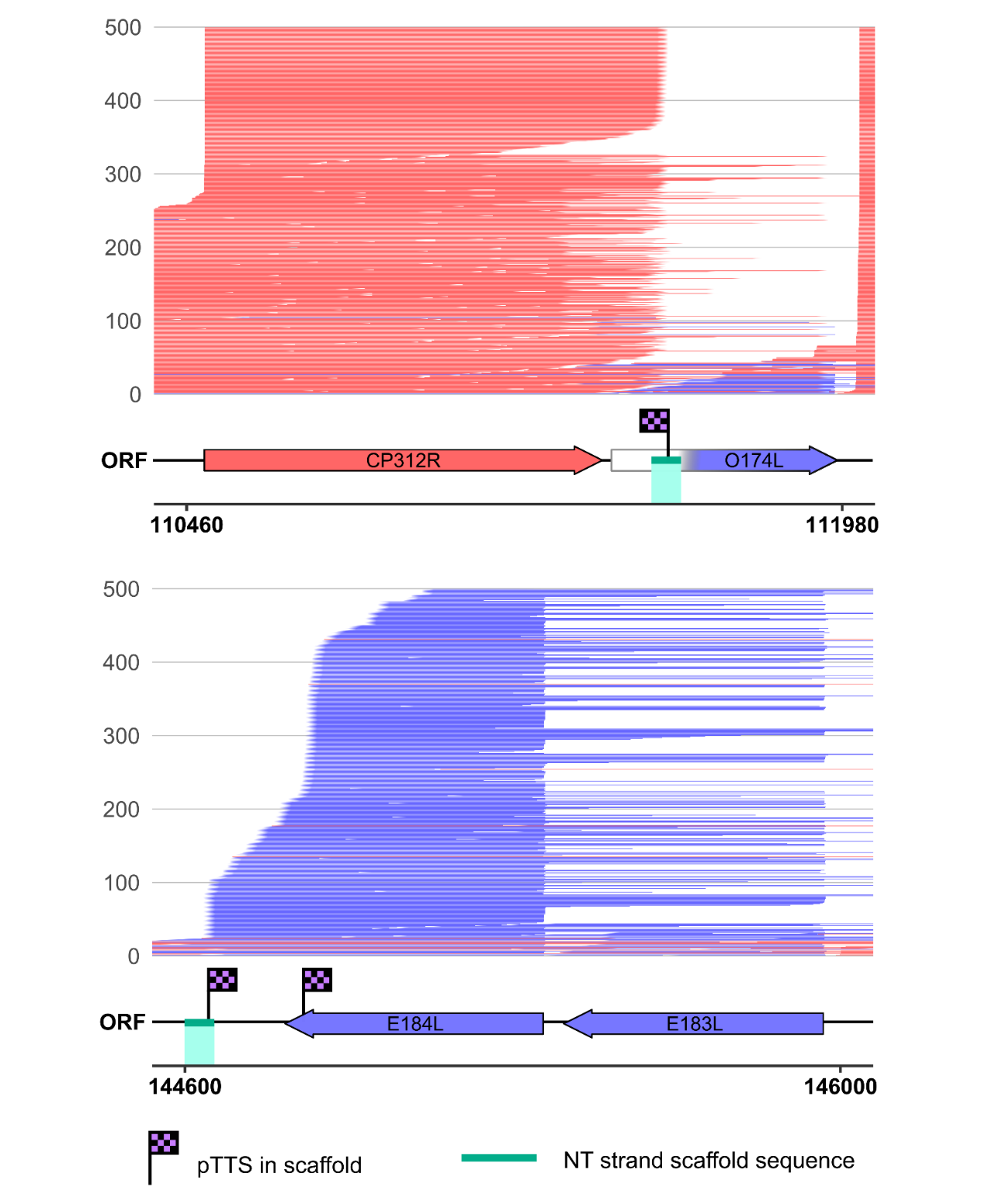


**Supplementary Figure 6.** Alignments of reads from 16 hpi for examples of polyT terminators for CP312R and E184L, the sequences of which (highlighted with sea green) were used for *in vitro* scaffolds in Figure 8.


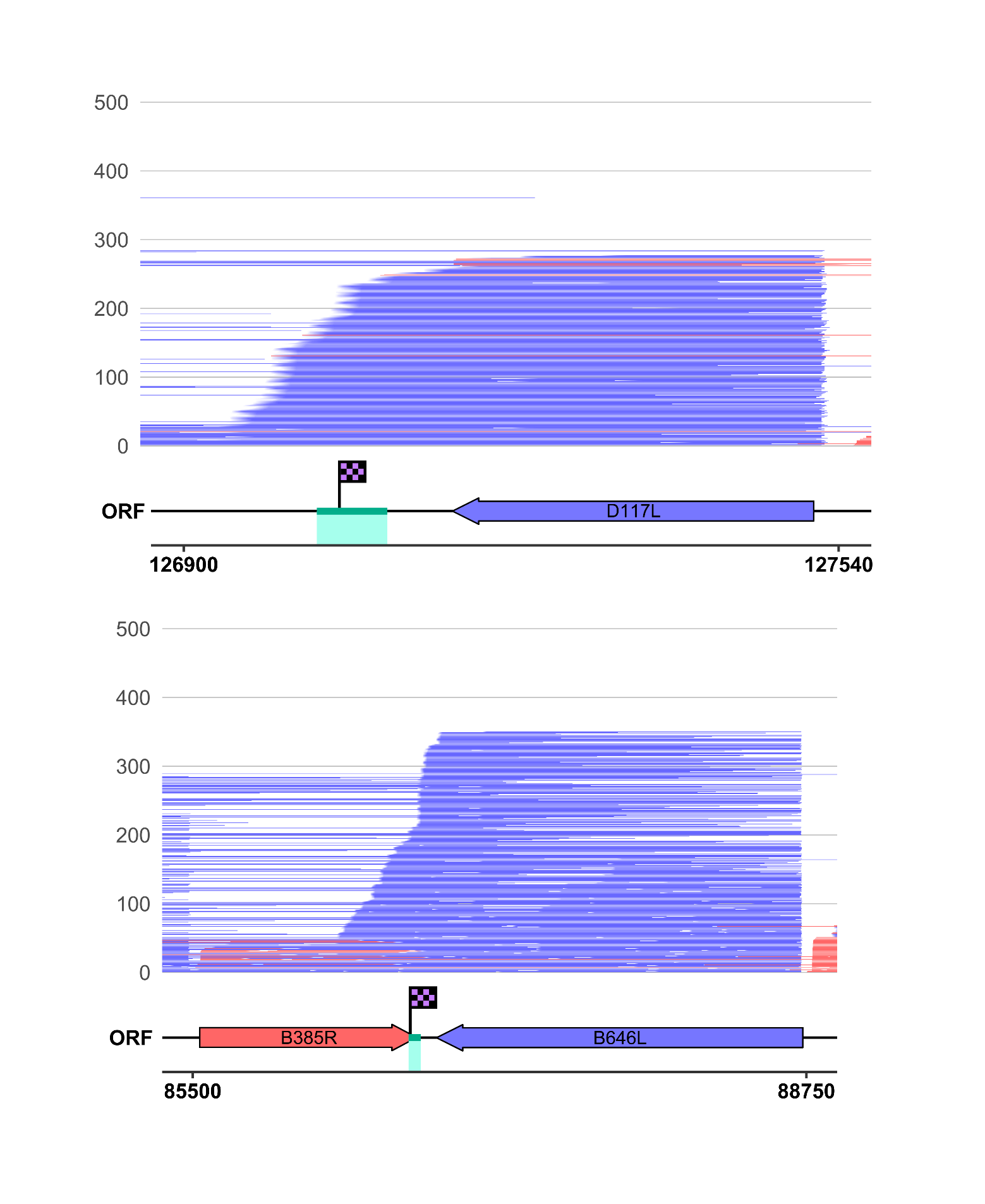


**Supplementary Figure 7.** Alignments of reads from 16 hpi for examples of polyA terminators for D117L and B646L, the sequences of which (highlighted with sea green) were used for *in vitro* scaffolds in Figure 8.


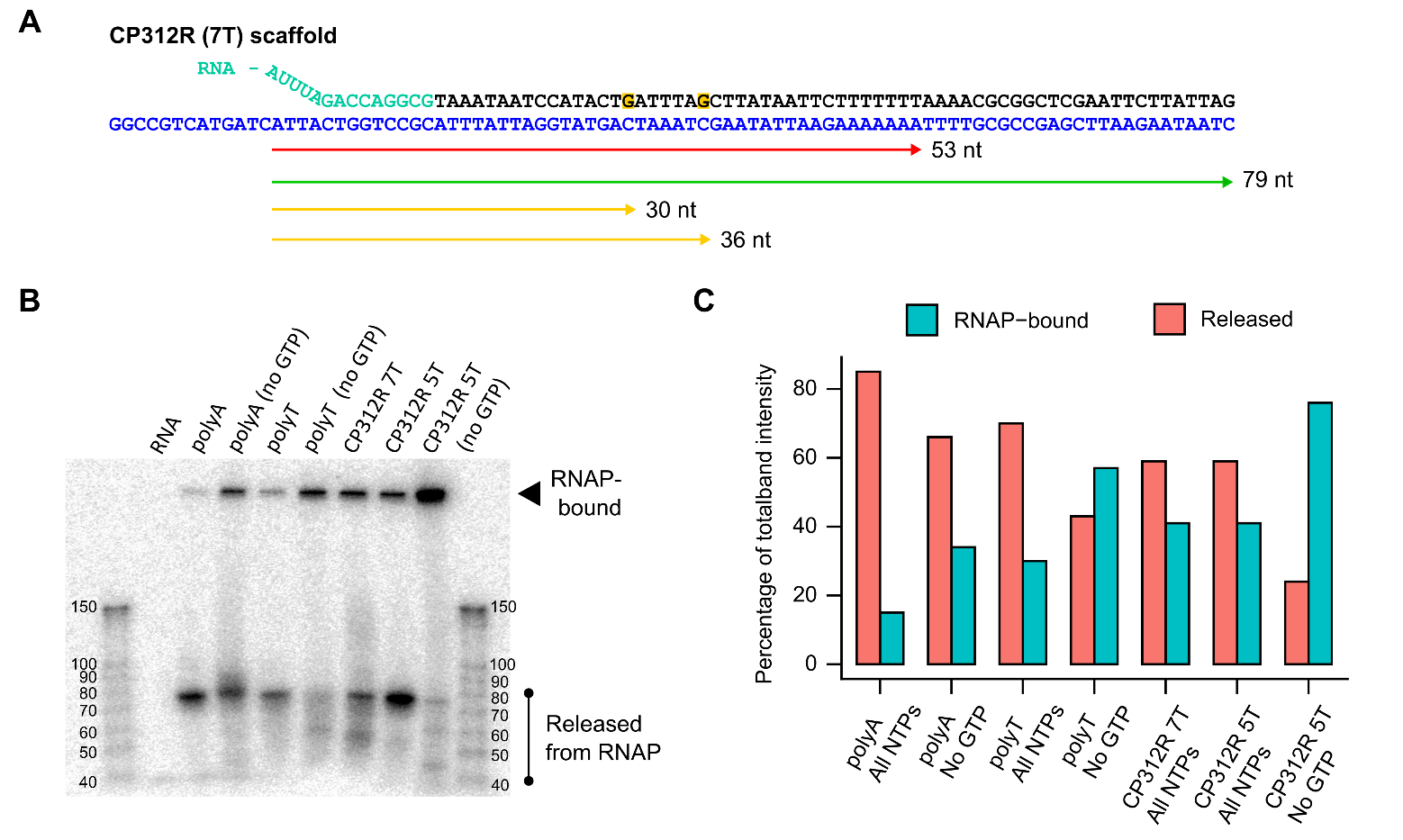


**Supplementary Figure 8.** (A) Detailed layout of CP312R scaffold sequence shown in Figure 8F. (B) *In vitro* transcription reactions as in Figure 8, run on TGX 4-15% gels under native conditions, using further ASFV and synthetic scaffolds. (C) Quantification of relative intensity for RNAP-bound versus released (as annotated in (B). Band intensity was estimated using GelAnalyzer (v. 23.1.1 available at www.gelanalyzer.com) by Istvan Lazar Jr., PhD and Istvan Lazar Sr., PhD, CSc.

1. Cackett, G. et al. The African Swine Fever Virus Transcriptome. *Journal of virology* **94**(2020).
